# Algorithm‐Based Common Microcirculatory Framework for Monitoring and Visualizing the Integrated Pancreatic Microcirculation in Type 2 Diabetes Mellitus Mice

**DOI:** 10.1111/1753-0407.70188

**Published:** 2026-02-04

**Authors:** Yuan Li, Yingyu Wang, Bing Wang, Weiqi Liu, Mengting Xu, Xiaoyan Zhang, Xueting Liu, Hao Ling, Xu Zhang, Mingming Liu, Ruijuan Xiu

**Affiliations:** ^1^ Institute of Microcirculation, Chinese Academy of Medical Sciences & Peking Union Medical College, Key Laboratory of Microcirculation, Ministry of Health Beijing China; ^2^ International Center of Microvascular Medicine Chinese Academy of Medical Sciences Beijing China; ^3^ Department of Radiology, the Affiliated Changsha Central Hospital, Hengyang Medical School University of South China Changsha China; ^4^ Laboratory of Electron Microscopy, Ultrastructural Pathology Center Peking University First Hospital Beijing China; ^5^ Diabetes Research Center Chinese Academy of Medical Sciences Beijing China

**Keywords:** computational framework, liraglutide, microhemodynamics, pancreatic microcirculation, type 2 diabetes mellitus

## Abstract

**Background:**

Recent research has challenged the viewpoint that pancreatic islets operate independently of surrounding exocrine tissues, revealing a bidirectional blood flow between the endocrine and exocrine pancreas. However, a methodology for simultaneous evaluation of pancreatic microhemodynamics and oxygen profiles remains elusive.

**Methods:**

To generate the common microcirculatory framework, we employed laser Doppler and diffuse reflectance spectroscopy to assess pancreatic microcirculation with concurrent acquisition of microhemodynamic and oxygen data as time‐series measurements. The framework's analytical pipeline, featuring outlier adjustment using the boxplot algorithm and comparative normalization strategies (*Z*‐score, min–max, L2, and median scaling), was subsequently validated in a T2DM mouse model with insulin and liraglutide‐administered groups. Heat maps and chord plots were used to reveal the integrated dynamics of the associations between microcirculatory blood perfusion and oxygen saturation.

**Results:**

The established common microcirculatory framework effectively characterized integrated microhemodynamics and oxygen profiles, with min–max normalizing the microhemodynamic and oxygen. T2DM mice exhibited decreased blood perfusion, reduced red blood cell tissue fraction, diminished oxygen saturation, and lower hemoglobin concentration within the pancreatic microcirculation. Treatment with liraglutide significantly ameliorated these microcirculatory impairments, partially restoring the balance between blood perfusion and oxygen saturation and normalizing the disrupted coherence between oxygenated hemoglobin and speed‐resolved blood perfusion.

**Conclusions:**

The common microcirculatory framework provides a novel methodology for monitoring, visualizing, and assessing integrated pancreatic microcirculatory function, with liraglutide demonstrating enhanced efficacy in ameliorating microcirculatory dysfunction in T2DM.

## Highlights

- A novel framework integrates concurrent microhemodynamic and oxygenation data to assess microcirculatory function.
- Liraglutide improved impairments in pancreatic blood perfusion, red blood cell tissue fraction, oxygen saturation, and hemoglobin concentration in pancreatic microcirculation under T2DM status.
- Liraglutide restores the physiological coupling between blood flow and oxygen availability in diabetic pancreatic microcirculation.

## Graphical abstract

Integrated monitoring of the pancreatic microcirculation utilizes a multimodal probe to simultaneously acquire microhemodynamic and oxygenation data through combined laser Doppler flowmetry and diffuse reflectance spectroscopy (DRS). Raw signals undergo algorithmic processing to extract speed‐resolved blood perfusion (BP) parameters, categorized into low, middle, and high‐speed components, alongside essential hemoglobin (Hb) metrics including red blood cell tissue fraction (C_RBC_), oxygen saturation (SO_2_), and oxygenated versus reduced hemoglobin levels. Data processing pipeline incorporates outlier adjustment using the boxplot algorithm followed by comparative normalization strategies, specifically Min‐Max and Z‐score scaling, to standardize the concurrent microcirculatory metrics. The computational pipeline facilitates the transformation of concurrent time‐series measurements into a standardized framework, enabling the visualization of disrupted microcirculatory coupling and the subsequent therapeutic response to pharmacological intervention in the diabetic pancreas.

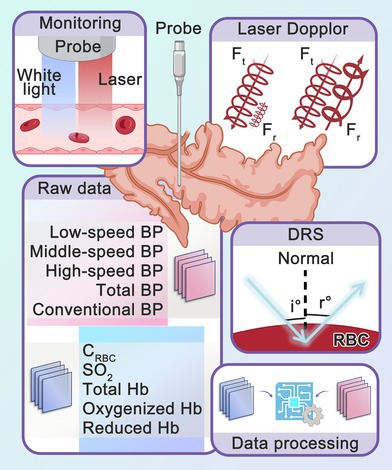

## 1 Introduction

The pancreatic microvasculature, composed of arterioles, venules, and capillaries less than 150 μm in diameter, creates a complex network that integrally connects the endocrine and exocrine components of the pancreas [1, 2]. While traditionally viewed as having independent blood flow, the network is now understood as a highly integrated system where the classic concept of a unidirectional insulo‐acinar portal system has been revised. Modern high‐resolution imaging has revealed a new paradigm of seamless vascular integration and bidirectional flow, transforming the relationship into a dynamic, two‐way dialogue between the two compartments [3, 4, 5]. The dynamic architecture is highly specialized; islet vasculature is characterized by high density and fenestrated endothelium to facilitate rapid hormone transport [6], whereas the exocrine vasculature is adapted for acinar cell metabolism. Disturbances in pancreatic microcirculation can exacerbate pathologies such as acute and chronic pancreatitis [7, 8], pancreatic ductal adenocarcinoma [9], and type 2 diabetes mellitus (T2DM) [10, 11].

The concept of metabolism‐matched microcirculatory blood perfusion is essential for comprehending tissue and organ function, as it ensures adequate oxygen supply and nutrient delivery, thus preserving cellular integrity and performance [12]. In T2DM, pathologies induce widespread damage that affects both compartments, and the failure of the perfusion matching is a core mechanism driving the disease's progression and its devastating multiorgan complications. Therefore, an assessment of the integrated microcirculatory state, which can account for the functional consequences of the bidirectional communication, rather than the function of isolated vascular domains, is essential for understanding the collective impact of systemic disease on the entire organ.

Investigative techniques, such as corrosion casting, contrast‐enhanced ultrasound [13, 14], optical coherence tomography angiography [15, 16, 17], and contrast‐enhanced magnetic resonance imaging [18, 19, 20], have provided the functional and structural aspects of the microvasculature. However, these methods often fall short in capturing the dynamic nature of microcirculatory function. For instance, while techniques like corrosion casting produce detailed three‐dimensional anatomical maps, they are terminal and static, providing no information on real‐time blood flow. Functional imaging modalities, on the other hand, typically provide time‐averaged perfusion data and lack the spatiotemporal resolution needed to resolve the rapid, second‐to‐second fluctuations that govern tissue homeostasis. A reliance on static representations of microvascular morphology or indirect assessments may lead to an incomplete understanding of the physiological and pathophysiological processes involved. Therefore, real‐time monitoring and visualization of microhemodynamic and oxygen profiles are critical for evaluating integrated pancreatic function.

To address these needs, we have developed a methodology that employs algorithms for real‐time monitoring and visualization of synchronized time‐series pancreatic microhemodynamics and oxygen profiles. Validation of this methodology has been conducted through analyses involving T2DM model mice treated with insulin and liraglutide. By elucidating the associations between microcirculatory dysfunction and metabolic regulation, the aim of our study is to establish a framework that captures this holistic functional status, providing a macro‐level view of pancreatic microcirculatory health and its response to therapeutic intervention.

## 2 Methods

### 2.1 Establishment of the Common Microcirculatory Framework

**FIGURE 1:**
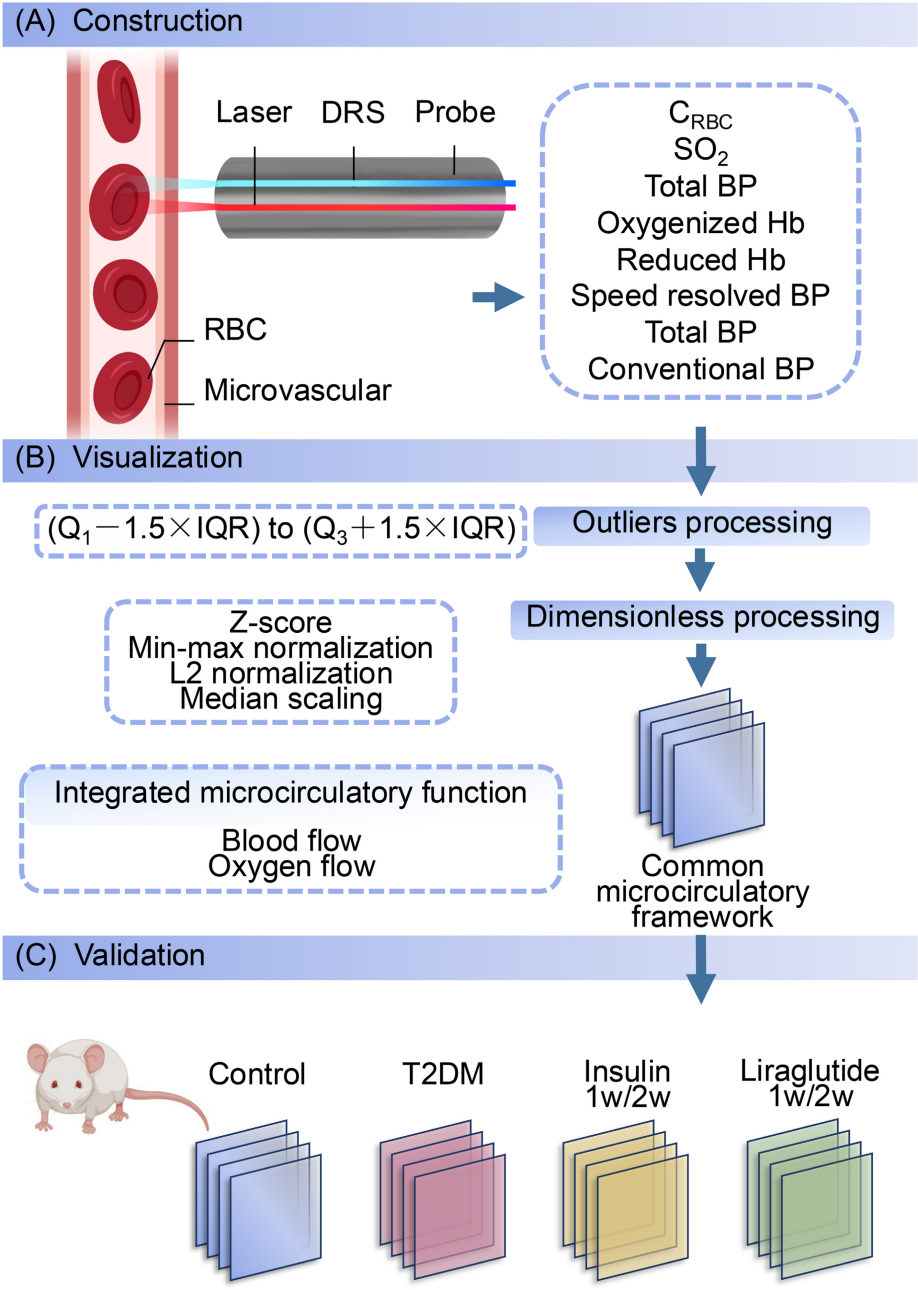
Schematic of the construction of common microcirculatory framework. (A) Establishment of common microcirculatory framework. The probe equipped with laser and DRS was located upon the exposed tissue. Subsequently, real‐time synchronal pancreatic microcirculatory oxygen and microhemodynamic data, including C_RBC_, SO_2_, total Hb, oxygenized Hb, reduced Hb, total BP, speed‐resolved BP, and conventional BP, were captured. (B) Visualization module. The outliers and dimensions of the raw microcirculatory data were eliminated and subsequently integrated as the common microcirculatory framework. (C) Validation module. For validation, microcirculatory function data of mice in control, T2DM, insulin‐administered (1 week/2 weeks), and liraglutide‐administered (1 week/2 weeks) groups were harvested and embedded in the established common microcirculatory framework. BP, blood perfusion; CRBC, red blood cell tissue fraction; DRS, diffuse reflectance spectroscopy; Hb, hemoglobin concentration; RBC, red blood cell; SO_2_, oxygen saturation; T2DM, type 2 diabetes mellitus.

The common microcirculatory framework was established using a combination of validated optical sensing technologies and algorithm‐based data integration modules to provide a holistic view of microvascular function (Figure 1). The core of the data acquisition relied on instrumentation equipped with laser Doppler flowmetry (LDF) and diffuse reflectance spectroscopy (DRS). These optical techniques are individually well‐established for real‐time assessment of microcirculation in highly vascularized organs and tissues [21, 22, 23]. Demo permutations of microcirculatory patterns were algorithmically generated through a computation process utilizing the characteristic features of microcirculatory oxygen and the microhemodynamics within biological tissues. Subsequently, the creation of the three‐dimensional (3D) framework was executed utilizing Python (version 3.7.4; Python Software, DE, USA) in conjunction with Apache ECharts (version 4.2.0‐RC.2; Apache Software, DE, USA). The temporal progression was represented along the *X*‐axis, while the multiple variables were defined along the *Y*‐axis, and the demo microcirculatory values were allocated to the *Z*‐axis.

### 2.2 Data Preprocessing and Signal Refinement

To ensure the integrity and accuracy of the raw, synchronous data streams, a computational pipeline was implemented, beginning with data preprocessing. Outliers are defined as values of exceptional magnitude that exhibit substantial deviation from the central tendency within the microcirculatory dataset, exhibiting a synchronous departure from the majority of observations (Figure 1B). In the preprocessing module, outliers were identified utilizing the boxplot algorithm. *Q*_1_ was defined as the 25th percentile upper value and *Q*_3_ as the 75th percentile upper value, thereby establishing the interquartile range (IQR) as the difference between *Q*_3_ and *Q*_1_. Any microcirculatory variables falling outside the range from (*Q*_1_–1.5 × IQR) to (*Q*_3_ + 1.5 × IQR) were identified as outliers and subsequently subjected to an automated procedure for adjustment to the nearest boundary value. The application of the standard and statistical method mitigates the influence of artifacts and enhances the overall signal fidelity before subsequent analysis. Additionally, the least common multiple algorithms were employed in guaranteeing uniformity in data dimensions within the framework.

### 2.3 Comparison of Four Dimensionless Methods

For the purpose of unifying the microcirculatory variables and achieving a comprehensive depiction of the comprehensive functional state of microcirculation, four common dimensionless techniques, namely *Z*‐score normalization, min–max normalization, L2 normalization, and median scaling, were applied to render the microcirculatory dataset dimensionless (Figure 1B).

*Z*‐score normalization, as a data normalization strategy, offers a means of data standardization that effectively mitigates the influence of outliers. The magnitude of “*z*” represents the extent to which the raw microcirculatory dataset deviates from its mean in standard deviation units. The equation dictating the data processing protocol for *Z*‐score normalization is articulated as follows:

(1) $$x^{\prime }=\frac{x-\bar{x}}{\sigma }$$

where x denotes the original microcirculatory variable. $\bar{x}$ signifies the mean value of the microcirculatory feature. *σ* is the standard deviation.

Min–max normalization, also referred to as deviation normalization, processes the dataset based on extreme values and classifies the processed data within the interval [0, 1]. The microcirculatory dataset was subjected to a transformation where the minimum value was normalized to 0, and the maximum value was scaled to 1. The expression for min–max normalization is as follows:

(2) $$dx^{\prime }=\frac{x-\min}{\max-\min}$$

where min and max are the minimum and maximum values of the microcirculatory dataset, respectively.

L2 normalization is a well‐established technique in the context of dimensionless normalization for multiple datasets, which quantifies the vector magnitude and adjusts the components of the feature vector accordingly. In this method, each microcirculatory component is normalized by the Euclidean vector magnitude. The formulation for L2 normalization can be expressed as follows:

(3) $${x_{i}}^{\prime }=\frac{x_{i}}{\mathrm{norm}\left(x\right)}$$

where $\mathrm{norm}\left(x\right)$ is as follows:

(4) $$\mathrm{norm}\left(x\right)=\sqrt{{x_{1}}^{2}+{x_{2}}^{2}+\ldots +{x_{n}}^{2}}$$

Median scaling represents the nonparametric method of standardization (centering and scaling) [24]. All microcirculatory datasets underwent normalization by dividing each data point by the median value. The dataset processing equation of median scaling is as follows:

(5) $$x^{\prime }=\frac{x}{\mathrm{Me}}$$

where Me is the median of the microcirculatory dataset.

### 2.4 Animals

The animal study was approved by the Institutional Animal Care and Use Committee (IACUC) of the Institute of Microcirculation, Chinese Academy of Medical Sciences (CAMS), and was conducted in accordance with the principles and guidelines of the Care and Use of Laboratory Animals (IACUC‐201709). Eight‐week‐old male BALB/c mice were procured from the Institute of Laboratory Animal Sciences (CAMS, Beijing, China) and housed under a 12:12 h light/dark cycle at 26°C and 55%–70% humidity.

### 2.5 Induction of T2DM, Administration of Insulin and Liraglutide

Mice were randomly divided into control (*n* = 9) and T2DM (*n* = 45) groups. To induce T2DM model, mice were fed with high‐fat diet (60% fat, 20% carbohydrate, and 20% protein diet; energy ratio, kcal %; D12492, HFK Bioscience, Beijing, China) for 4 weeks, and then streptozotocin (STZ; Sigma‐Aldrich, Darmstadt, Germany; 50 mg/kg in 0.1 mol/L citrate buffer, pH = 4.3) was injected via intraperitoneal route in two consecutive days (Figure S1). To avoid hypoglycemia, glucose saline solution (Sigma‐Aldrich; 0.2 g/kg) was injected intraperitoneally within 6 h after each STZ injection with 10% sucrose (Sigma‐Aldrich), provided in drinking water for 2 days. Twelve days after the STZ injection, the One Touch UltraEasy glucometer (Johnson and Johnson, CA, USA) was employed to measure the tail vein blood samples for fasting blood glucose (FBG) assessment, by which the hyperglycemia of T2DM was confirmed in mice with FBG exceeding 200 mg/dL (Figure S2). Thirty‐six of the confirmed T2DM mice were then divided into insulin‐ and liraglutide‐treated subgroups and were administered with insulin (Humalog Mix25, Eli Lilly, IN, USA; 1 IU, *i.p*.) or liraglutide (Novo Nordisk, Copenhagen, Denmark; 0.2 mg/kg, *i.p*.) for 1 and 2 weeks, respectively.

### 2.6 Determination of Integrated Pancreatic Microcirculatory Profile

To evaluate the microcirculatory oxygen and microhemodynamics, an enhanced perfusion and oxygen saturation (EPOS) system (PF6000, Perimed AB, Stockholm, Sweden) was employed according to the previous protocol [25, 26]. The system performs a probe‐based, volumetric assessment of microcirculatory function. The probe's detection depth of approximately 1 mm means that the acquired signals represent the average functional status of a mixed tissue volume, which includes capillaries distributed across both endocrine islet and exocrine acinar tissues. The employed techniques generate time‐series data reflecting the average physiological state within a defined tissue volume, rather than providing spatial images of the microvasculature. Multiple parameters of the integrated microcirculation were measured using DRS and LDF (Figure 1A).

In brief, after a 30‐min acclimatization period, 2% isoflurane (RWD Life Science, Shenzhen, China) inhalation was used for anesthesia through an animal anesthesia apparatus (Matrx VMR, Midmark Corporation, OH, USA) supplied with a 50% oxygen mixture. Subsequently, a midline incision was made to expose the pancreas, and the detection probe was positioned upon the exposed pancreas using a probe holder. Real‐time synchronal pancreatic microcirculatory oxygen and microhemodynamic data, including the red blood cell tissue fraction (C_RBC_, %), oxygen saturation (SO_2_, %), total hemoglobin concentration (total Hb, μM), oxygenized hemoglobin concentration (oxygenized Hb, μM), reduced hemoglobin concentration (reduced Hb, μM), speed‐resolved perfusion (BP, %RBC × mm/s), total perfusion (total BP, %RBC × mm/s), and conventional perfusion (conventional BP, PU) were determined by EPOS (Table S1). The depth of the detective was approximately 1 mm. The divergence of microhemodynamic parameters was analyzed. Besides that, the index of pancreatic microcirculatory resistance (IMR_pancreas_) was set up and calculated as follows:

(6) $${\mathrm{IMR}}_{\text{pancreas}}=\frac{\mathrm{Mbp}}{\text{Mvel}\times \text{Mfre}\times S}$$

where “IMR_pancreas_” represents the index of pancreatic microcirculatory resistance, “Mbp” means the microvascular blood perfusion, “Mvel” is the microcirculatory blood perfusion velocity, “Mfre” refers to the number of microcirculatory blood perfusion oscillations per minute, while “*S*” represents the diameter of microvasculature and is defined as a constant (150 μm) [27]. The “Mvel” was calculated as follows [28]:

(7) $$\text{Mvel}=\frac{\mathrm{Mbp}}{\text{CRBC}}$$

where “C_RBC_” is the fraction of the sampling volume that consists of red blood cells.

### 2.7 Microcirculatory Functional Blotting

To visualize the distribution pattern of microcirculatory oxygen and microhemodynamics, the microcirculatory functional data were illustrated in the form of bubbles of different sizes. Different colors were used to distinguish among the six groups. The horizontal location gave information about the functional annotation of microcirculatory oxygen and microhemodynamics.

### 2.8 Microhemodynamic‐Oxygenic Correlation Analysis

To further investigate the correlation of microhemodynamic‐microcirculatory oxygenic profile among control, T2DM, and liraglutide‐treated mice, the chordal graph was subsequently generated by Dycharts (Dydata technology, Hubei, China). In the chordal graph, the left and right half arcs represent the levels of microcirculatory blood perfusion and SO_2_, respectively. According to the ranges of measured microcirculatory values, each half arc was divided into 10 intervals. The arc width represents the proportion of microcirculatory SO_2_ and blood perfusion. Arcs were connected by chords reflecting the microhemodynamic‐oxygenic consistency between microcirculatory SO_2_ and blood perfusion. Furthermore, the cluster correlations among the multiple microcirculatory parameters were generated by RStudio (version 8.02; RStudio, MA, USA). The relevance was attributed to results where both *p* < 0.05 and correlation coefficient values met the criteria of *r* > 0.4 or *r* < −0.4.

### 2.9 Video Recording

We utilized ScreenToGif software (version 2.19.3; Nicke Manarin, Brazil) to capture dynamic visualizations of the standardized common microcirculatory framework, which was processed with four dimensionless methods mentioned above. Moreover, the pancreatic microcirculatory frameworks of the control, T2DM, insulin‐, and liraglutide‐administered groups were recorded. Videos were stored in MP4 format with 1466 × 804 resolution.

### 2.10 Statistical Analysis

Statistical analyses were performed employing GraphPad Prism software (version 8.02; GraphPad Software, CA, USA). All the microcirculatory data were expressed as means ± standard error of the mean (SEM). The datasets underwent a *t*‐test between two groups, and a two‐way analysis of variance (ANOVA) was conducted to facilitate multiple group comparisons.

## 3 Results

### 3.1 Establishment of Common Microcirculatory Framework and Comparison of Four Dimensionless Methods

To characterize the integrated microcirculatory profiles, a common microcirculatory framework was designed, including modules of monitoring, data processing, and visualization (Figure 1). A demo dataset was generated based on the microcirculatory functional dataset of biological tissues. After outlier adjustment and processing, the multiple microcirculatory parameters were output into the visualization module to feed and establish the common microcirculatory framework.

**FIGURE 2:**
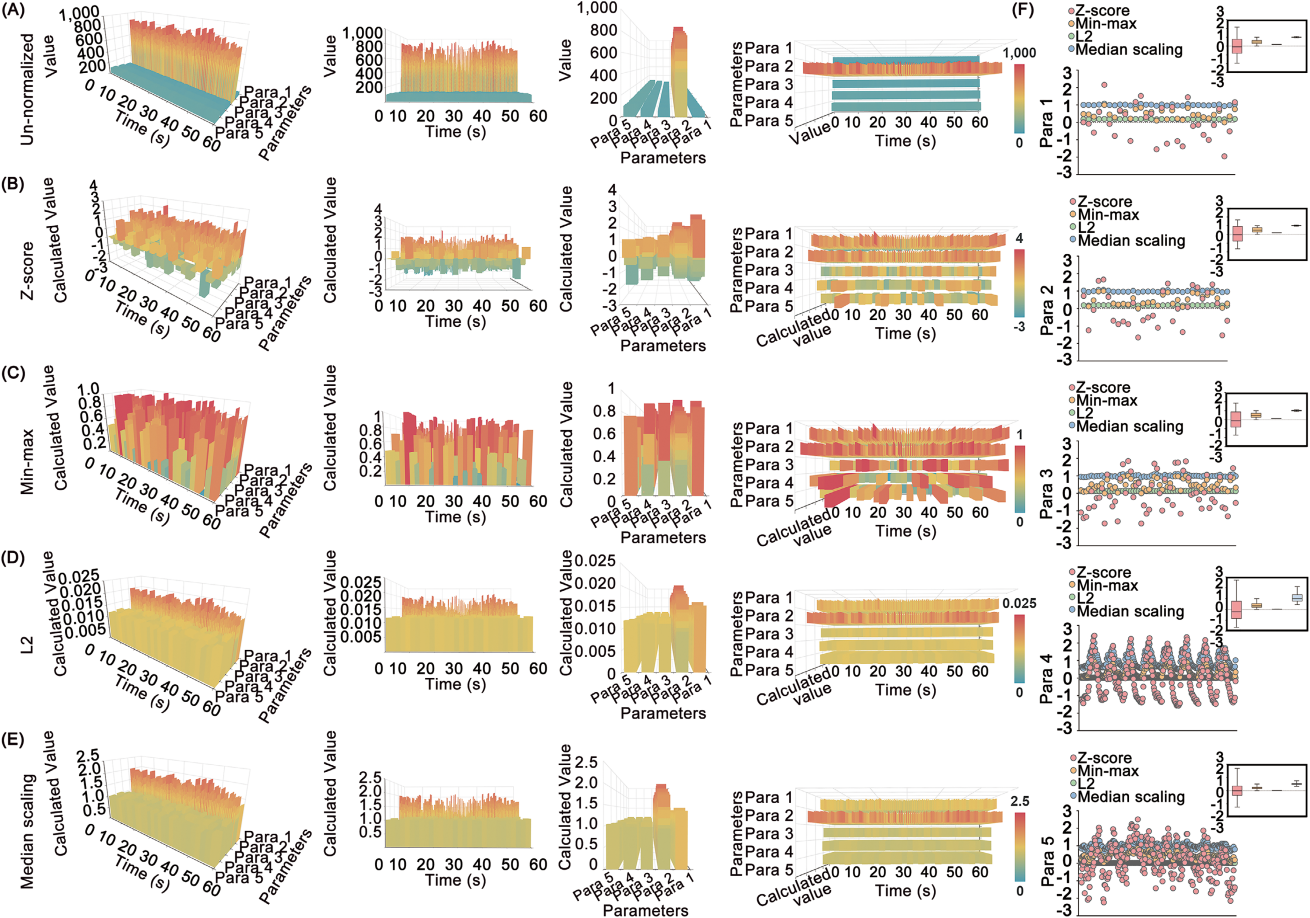
Comparison of four dimensionless methods in common microcirculatory framework. (A) Un‐normalized framework. Color bars represented the real values of the dataset. (B) *Z*‐score normalization processed framework. (C) Min–max normalized framework. (D) L2 normalized framework. (E) Median scaling normalized framework. (F) Capabilities of convergence and dispersion. The boxplots encompassed values ranging from the 25th percentile to the 75th percentile, and within each box, the median values were denoted by black lines. The whiskers extended to denote the minimum and maximum values for each normalized variable.

As shown in Figure 2A, unnormalized variables were severely compressed due to the dominant microcirculatory data. To comprehensively incorporate and visualize the characteristics of microcirculatory function, we subsequently adopted four dimensionless techniques that are widely employed in mathematical and computational investigations. These methods encompass *Z*‐score, min–max, L2 normalization, and median scaling, in which min–max normalization eliminated the differences in numerical size and parametric unit and contained fixing intervals during the analysis of multiple microcirculatory variables (Figure 2B–E). Moreover, the distribution trend of the multiple microcirculatory datasets was revealed after the *Z*‐score or min–max normalization (Figure 2F). Taken together, it was determined that min–max normalization was a more suitable approach for integrating and visualizing multiple microcirculatory variables within the common microcirculatory framework.

### 3.2 Liraglutide‐Induced Restoration of Pancreatic Microhemodynamics

**FIGURE 3:**
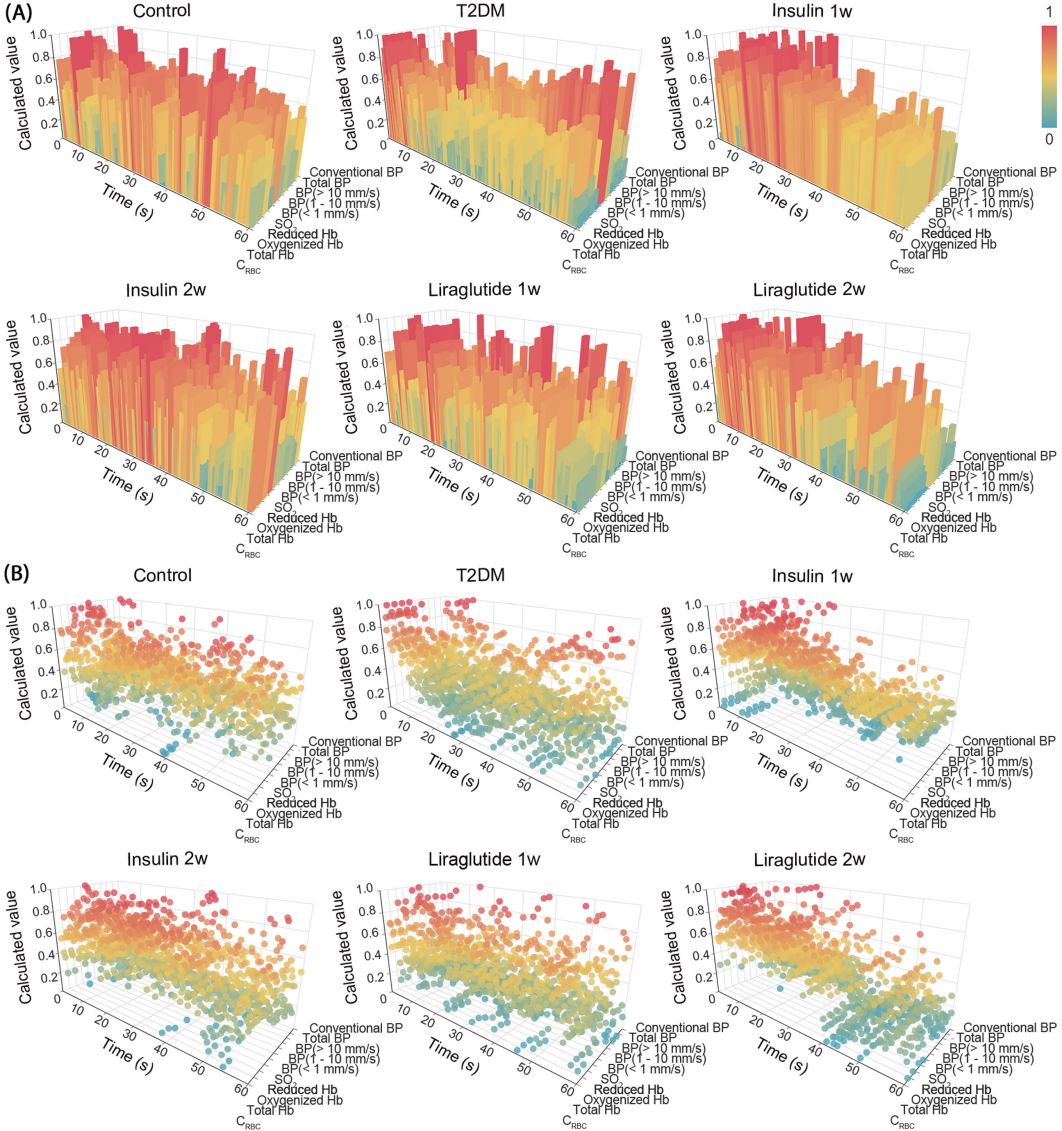
3D module of integrated microcirculatory profiles in pancreas. (A) Pancreatic microcirculatory oxygen profiles (C_RBC_, SO_2_, total Hb, oxygenized Hb, and reduced Hb) in side views (rotated 45° toward left from the front view). (B) The microhemodynamic profiles (speed‐resolved BP, total BP, and conventional BP) in side views. The *X*‐axis represented the temporal progression, the *Y*‐axis represented the microcirculatory parameters, and the *Z*‐axis denoted the calculated microcirculatory values. The color bar depicted the calculated values. 3D, three‐dimensional; BP, blood perfusion; CRBC, red blood cell tissue fraction; Hb, hemoglobin concentration; insulin 1w, 1‐week insulin‐administered T2DM group; insulin 2w, 2‐week insulin‐administered T2DM group; liraglutide 1w, 1‐week liraglutide‐administered T2DM group; liraglutide 2w, 2‐week liraglutide‐administered T2DM group; SO_2_, oxygen saturation; T2DM, type 2 diabetes mellitus.

**FIGURE 4:**
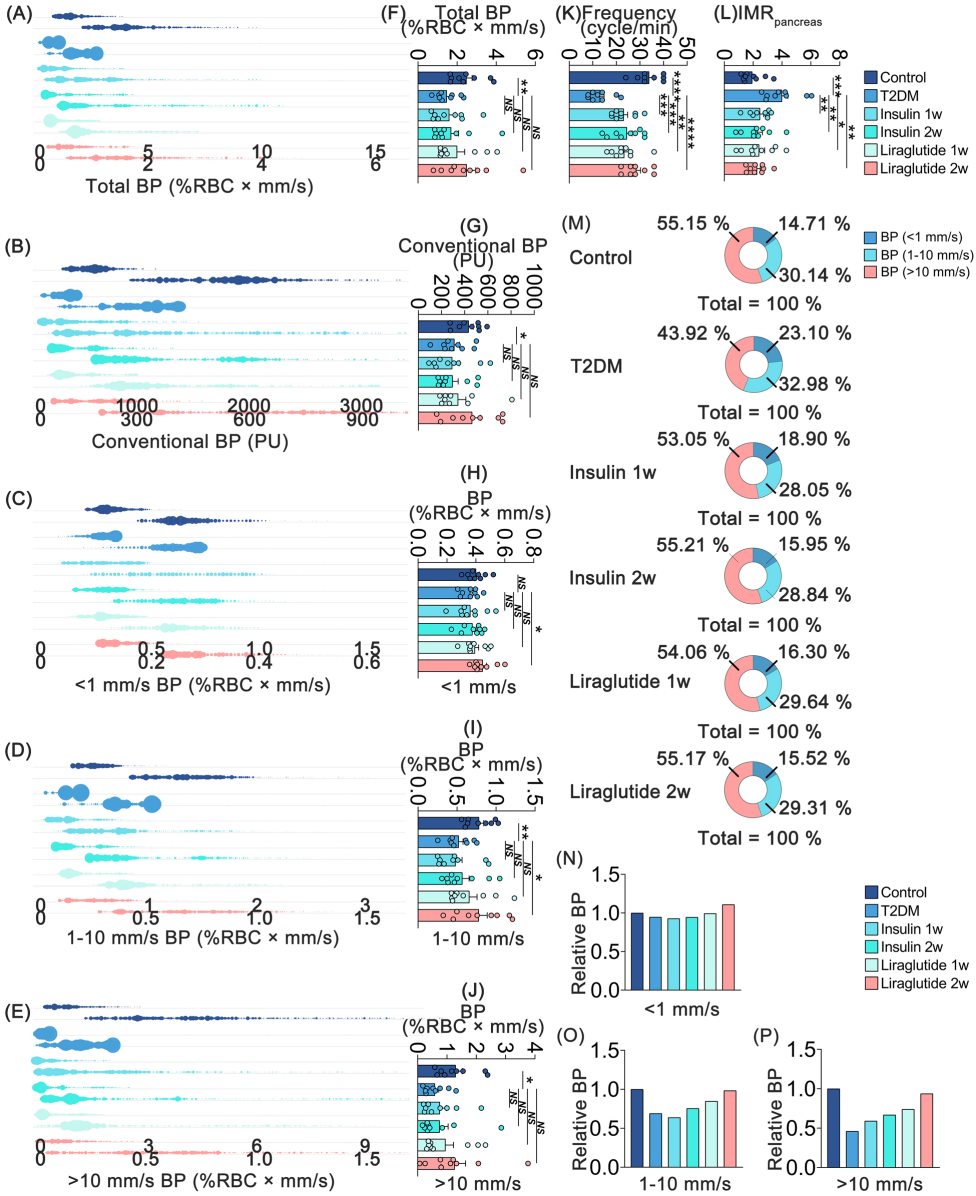
Comparisons of microhemodynamic profiles. (A–E) The distribution pattern of microhemodynamic profiles in biaxial bubble charts. (A) Total blood perfusion. (B) Conventional blood perfusion. (C) Low‐speed (< 1 mm/s) blood perfusion. (D) Mid‐speed (1–10 mm/s) blood perfusion. (E) High‐speed (> 10 mm/s) blood perfusion. (F–L) The levels of microhemodynamic profiles in histogram charts. (F) Total blood perfusion. (G) Conventional blood perfusion. (H) Low‐speed (< 1 mm/s) blood perfusion. (I) Mid‐speed (1–10 mm/s) blood perfusion. (J) High‐speed (> 10 mm/s) blood perfusion. (K) Frequency. (L) IMR_pancreas_. (M) Contributions of blood perfusion in three speed regions. (N–P) The levels of relative blood perfusion. (N) Relatively low‐speed (< 1 mm/s) blood perfusion. (O) Relative mid‐speed (1–10 mm/s) blood perfusion. (P) Relative high‐speed (> 10 mm/s) blood perfusion. Values are reported as means ± standard error of the mean (SEM). NS, no significant difference, *p < 0.05 and **p < 0.01. BP, blood perfusion; IMRpancreas, index of pancreatic microcirculatory resistance; insulin 1w, 1‐week insulin‐administered T2DM group; insulin 2w, 2‐week insulin‐administered T2DM group; liraglutide 1w, 1‐week liraglutide‐administered T2DM group; liraglutide 2w, 2‐week liraglutide‐administered T2DM group; T2DM, type 2 diabetes mellitus. *n* = 9 per group.

Next, to verify the applicability of the common microcirculatory framework, we embedded the dataset of T2DM and liraglutide‐administered groups into the microcirculatory framework (Figure 3 and Figures S3–S8, Video S1–S12). Compared with the control group, a statistical decrease in total blood perfusion was observed in T2DM mice (Figure 4A,F and Table S2), while a significant increase was found after liraglutide administration. Additionally, to further evaluate the microhemodynamics, the total blood perfusion was divided into three different speed regions: speeds below 1 mm/s (low‐speed perfusion), at 1–10 mm/s (mid‐speed perfusion), and above 10 mm/s (high‐speed perfusion). As illustrated in Figure 4C–E,H–J, significant decreases were observed in all three speed‐resolved blood perfusions in mice with T2DM, especially in high‐speed (> 10 mm/s) perfusions. The decreased blood perfusion can be partially reversed by liraglutide. Then we further analyzed the heterogeneity of the three speed‐resolved blood perfusion among groups and found a reduction (11.23%) in high‐speed (> 10 mm/s) perfusion and an increase (8.39%) in low‐speed (< 1 mm/s) perfusion in T2DM mice (Figure 4M). Liraglutide balanced the microcirculatory blood distribution in all three speed regions (Figure 4N–P). Additionally, T2DM mice exhibited a decrease in pancreatic microvascular frequency (Figure 4K) and an increase in IMR_pancreas_ (Figure 4L), both of which were partially restored following liraglutide administration. Collectively, these findings provide evidence that liraglutide improves pancreatic microhemodynamics in T2DM.

### 3.3 Improvement of Microcirculatory Oxygen in T2DM Through Liraglutide Administration

**FIGURE 5:**
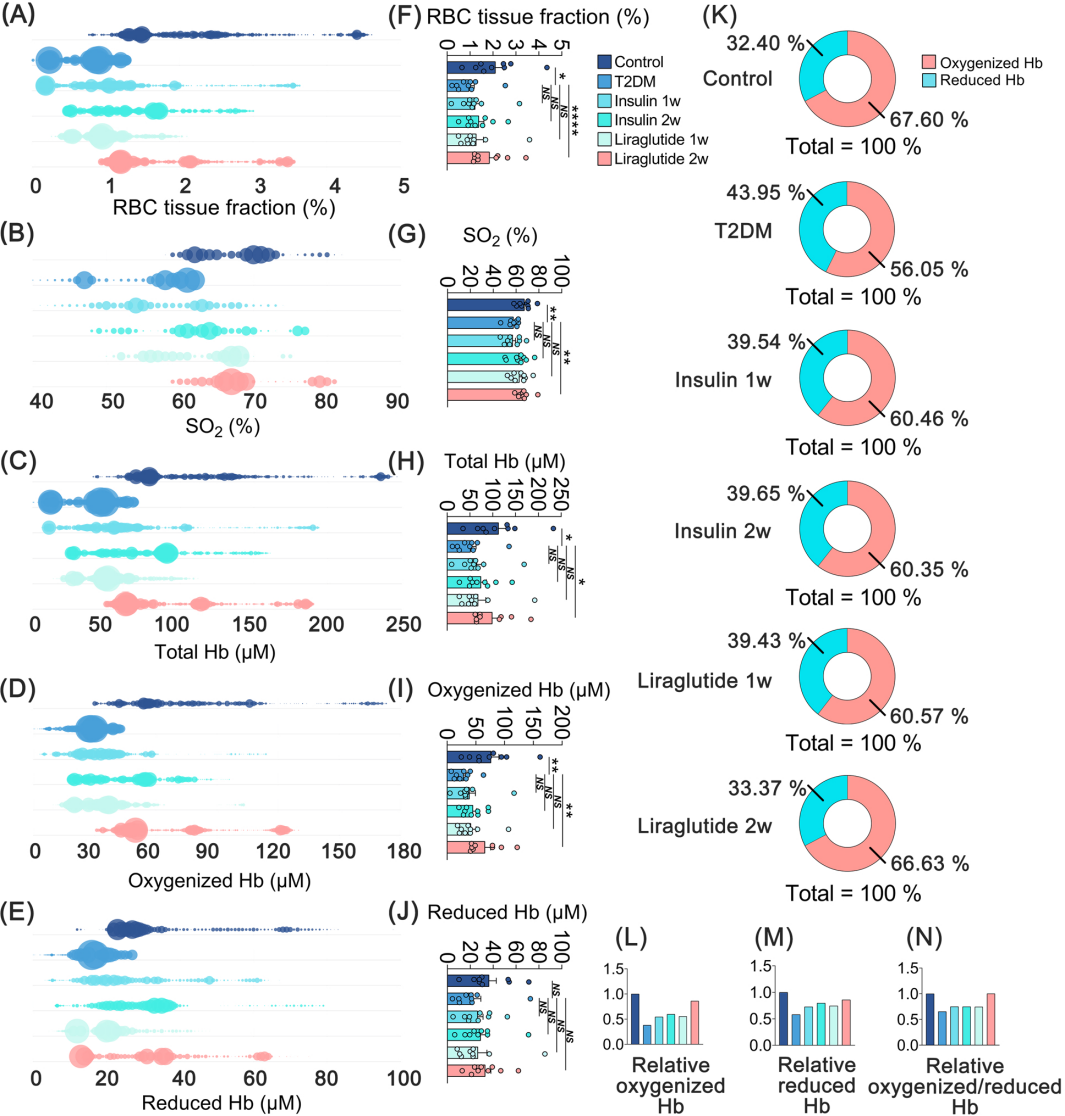
Comparisons of microcirculatory oxygen profiles. (A–E) The distribution pattern of microcirculatory oxygen profiles in bubble charts. (A) RBC tissue fraction. (B) SO_2_. (C) Total Hb. (D) Oxygenized Hb. (E) Reduced Hb. (F–J) The levels of microcirculatory oxygen profiles are shown in histogram charts. (F) RBC tissue fraction. (G) SO_2_. (H) Total Hb. (I) Oxygenized Hb. (J) Reduced Hb. (K) Contributions of oxygenized Hb and reduced Hb in total Hb. (L) The levels of relative oxygenized Hb. (M) The levels of relative reduced Hb. (N) The levels of relative oxygenized/reduced Hb. Values are reported as means ± standard error of the mean (SEM). NS, no significant difference, *p < 0.05, **p < 0.01, and ****p < 0.0001. Hb, hemoglobin concentration; insulin 1w, 1‐week insulin‐administered T2DM group; insulin 2w, 2‐week insulin‐administered T2DM group; liraglutide 1w, 1‐week liraglutide‐administered T2DM group; liraglutide 2w, 2‐week liraglutide‐administered T2DM group; RBC, red blood cell; SO_2_, oxygen saturation; T2DM, type 2 diabetes mellitus. *n* = 9 per group.

We then analyzed the pancreatic microcirculatory oxygen profiles, including the levels of red blood cell tissue fraction (C_RBC_, %), oxygen saturation (SO_2_, %), and hemoglobin (Hb, μM) concentration (total Hb, oxygenized Hb, and reduced Hb) dataset. The C_RBC_ represented the fraction of the sampling volume that consists of RBC, while the SO_2_ reveals the C_RBC_ in the sampling volume that is saturated. As shown in Figure 5A–J, compared with the control, C_RBC_, SO_2_, and Hb were significantly decreased in the T2DM group, indicating the presence of microcirculatory hypoxia, which could be alleviated by liraglutide administration. To further scrutinize the composition of microcirculatory Hb, the distribution of oxygenized Hb and reduced Hb was depicted by pie chart. In contrast to the control, the group with T2DM exhibited an 11.55% decrease in oxygenized Hb proportion (Figure 5K), suggesting the relative anoxic state in T2DM. Inversely, liraglutide administration increased in both relative oxygenized Hb and reduced Hb, as well as their ratio (Figure 5L–N). Liraglutide administration made increases in both relative oxygenized Hb and reduced Hb, as well as their ratio (Figure 5L–N). These findings demonstrated that liraglutide restores the pancreatic microcirculatory hypoxic state in T2DM.

### 3.4 Restoration of Microcirculatory Coherence Through Liraglutide in T2DM Mice

**FIGURE 6:**
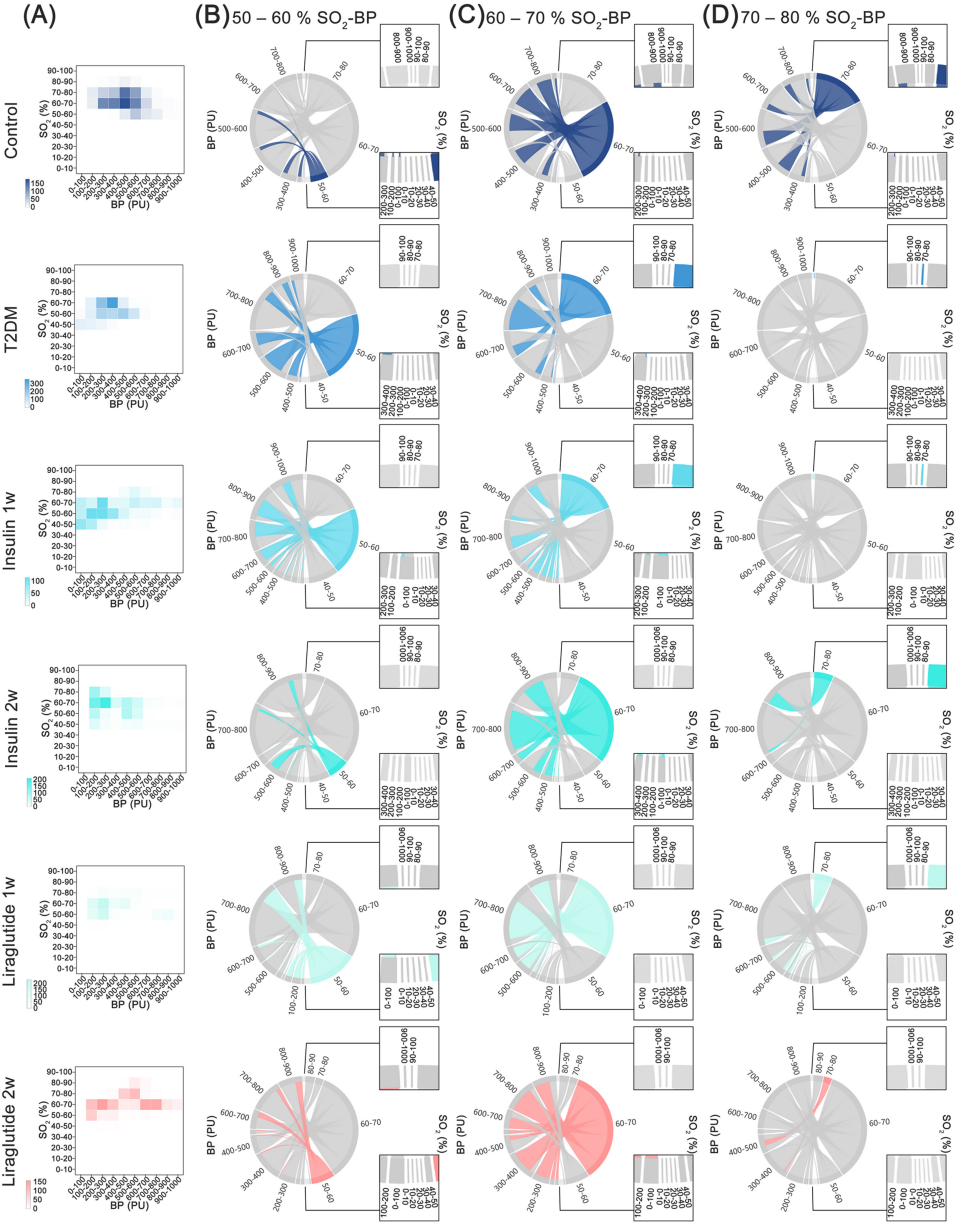
Microhemodynamic‐oxygenic consistency in different durations. (A) Heat map of the number of associations between microcirculatory blood perfusion and SO_2_. (B–D) Microhemodynamic‐oxygenic consistency in 50%–60% (B), 60%–70% (C), and 70%–80% (D) SO_2_ levels. The left and right half arcs represented the levels of microcirculatory blood perfusion and SO_2_, respectively. According to the microcirculatory function levels, each arc was assigned to 10 intervals. The arc width represented the amount of microcirculatory blood perfusion and SO_2_ data, and the chord width reflected the correlation attribute between blood perfusion and SO_2_. BP, blood pressure; insulin 1w, 1‐week insulin‐administered T2DM group; insulin 2w, 2‐week insulin‐administered T2DM group; liraglutide 1w, 1‐week liraglutide‐administered T2DM group; liraglutide 2w, 2‐week liraglutide‐administered T2DM group; SO_2_, oxygen saturation; T2DM, type 2 diabetes mellitus.

SO_2_ guided the distribution of oxygen‐carrying RBCs, reflecting the blood perfusion reserves in microcirculation. Owing to the synchronous acquisition of microhemodynamic and microcirculatory oxygen parameters, the data derived from these two dimensions exhibit temporal concordance. Therefore, we next aimed to explore the relationship between pancreatic microcirculatory oxygen and microhemodynamics by investigating the coherence of microcirculatory SO_2_–blood perfusion through network analysis (Figure 6 and Figure S9). Frequency heat matrix determined that the modes of the SO_2_–blood perfusion associations fell within the 50%–80% SO_2_ interval (Figure 6A). Subsequent analysis revealed that the blood perfusion primarily maintained the 60%–70% SO_2_ level in the healthy controls (Figure 6C). On the contrary, the blood perfusion in the T2DM group was mainly to sustain a lower (50%–70%) SO_2_ level (Figure 6B,C). Moreover, in the T2DM group, pancreatic microcirculation mobilized more blood perfusion to sustain the same (50%–70%) SO_2_ level (Figure 6B,C), but could not meet higher (> 70%) SO_2_ level as the control group (Figure 6D). These features suggested that the compensatory response in the microhemodynamic pattern could not sustain enough SO_2_ in pancreatic microcirculation in T2DM status. As expected, treatment by insulin and liraglutide (insulin administration for 2 weeks and liraglutide administration for 1 and 2 weeks) partially restored the blood perfusion for high (> 70%) SO_2_ level (Figure 6D) and reconstructed the deteriorative SO_2_–blood perfusion coherence (Figure 6B,C).

**FIGURE 7:**
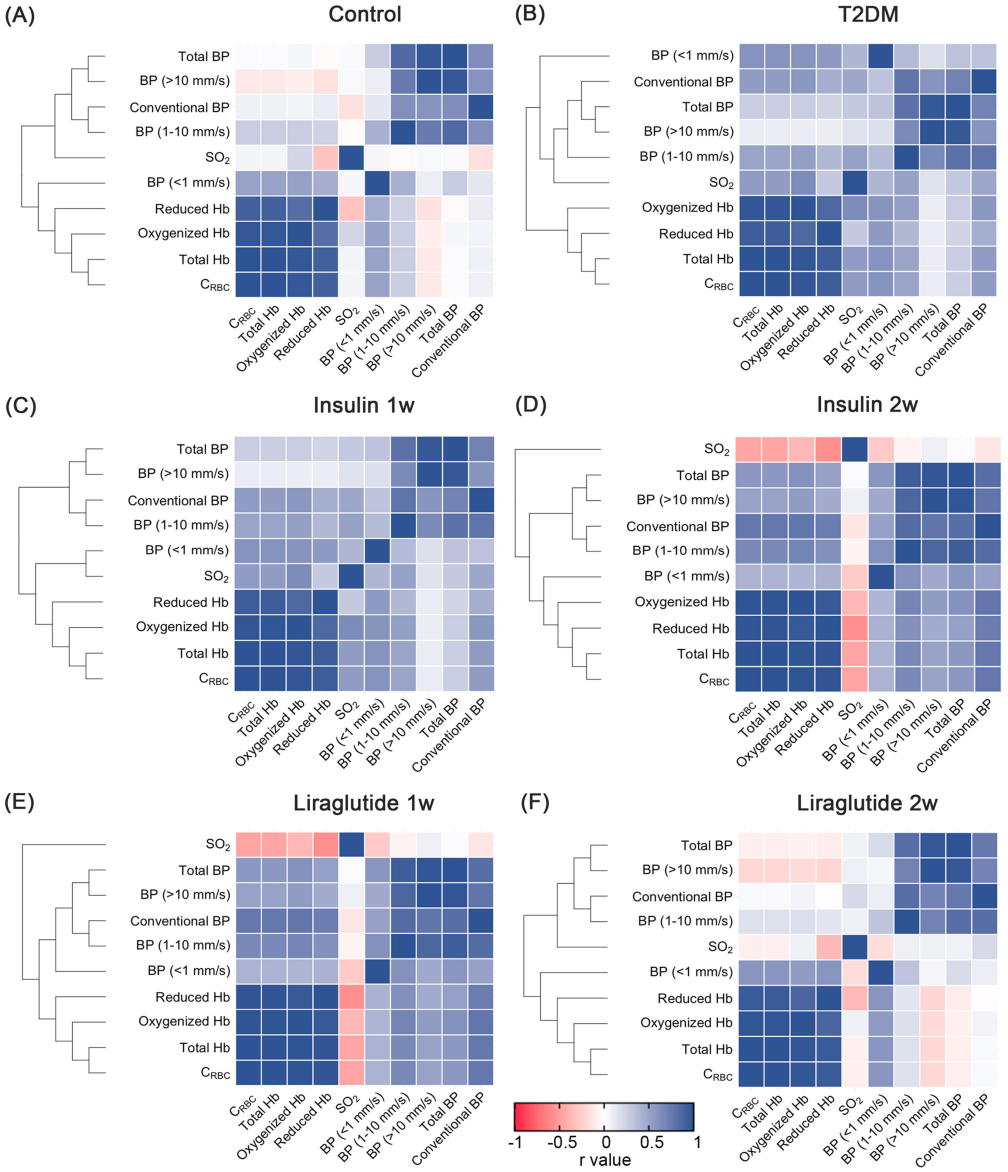
Hierarchical clustering analysis among microcirculatory oxygen and microhemodynamic profile. (A–F) Heat map of microcirculatory oxygen (C_RBC_, SO_2_, total Hb, oxygenized Hb, and reduced Hb) and microhemodynamic profile (speed‐resolved BP, total BP, and conventional BP). The blue color represented a positive correlation, while red represents a negative correlation. The cluster relationships are shown as the connecting lines on the left. (A) Control group. (B) T2DM group. (C) Insulin 1‐week administered group. (D) Insulin 2‐week administered group. (E) Liraglutide 1‐week administered group. (F) Liraglutide 2‐week administered group. *p < 0.05, **p < 0.01, ***p < 0.001, and ****p < 0.0001. BP, blood perfusion; CRBC, red blood cell tissue fraction; Hb, hemoglobin concentration; insulin 1w, 1‐week insulin‐administered T2DM group; insulin 2w, 2‐week insulin‐administered T2DM group; liraglutide 1w, 1‐week liraglutide‐administered T2DM group; liraglutide 2w, 2‐week liraglutide‐administered T2DM group; NS, no significant difference; SO_2_, oxygen saturation; T2DM, type 2 diabetes mellitus.

To further explore the relationship between microcirculatory oxygen and microhemodynamics, especially in different speed‐resolved blood perfusion, a clustering heat map was illustrated. As shown in Figure 7A,B, the oxygenized Hb in T2DM group exhibited weaker positive correlations with low‐speed (< 1 mm/s) (control: *r* = 0.520, *p* < 0.0001; T2DM: *r* = 0.323, *p* < 0.0001) and mid‐speed (1–10 mm/s) perfusion (control: *r* = 0.273, *p* < 0.0001; T2DM: *r* = 0.072, *p* = 0.0058) when comparing with the controls, respectively. Meanwhile, negative correlation was noticed between oxygenized Hb and high‐speed (> 10 mm/s) perfusion (control: *r* = −0.090, *p* = 0.0028; T2DM: *r* = −0.085, *p* = 0.001). Liraglutide administration effectively normalized the abnormal correlations between oxygenized Hb and three speed regions (Figure 7E,F). In addition, frequency clustered in control showed a negative correlation with IMR_pancreas_, while no significant correlation was found in the T2DM group (Figure S10). Besides that, liraglutide positively correlated with the negative relationship between microvascular frequency and IMR_pancreas_. Taken together, our results provide evidence that liraglutide may partly regain the coherence between microcirculatory oxygen and the microhemodynamics of the pancreatic microcirculation.

## 4 Discussion

The microvascular network orchestrates a symphony of physiological processes, with microhemodynamics and tissue oxygenation as pivotal components that govern the efficacy of microcirculatory function [29, 30]. In our study, we developed a common microcirculatory framework to simultaneously analyze the pancreatic microhemodynamics and oxygen parameters. A challenge is the quantitative integration of multi‐parameter microcirculatory data, which our analytical framework was designed to overcome [31]. The framework ensures high signal fidelity, beginning with the algorithmic correction of outliers that arise from technical non‐biological variations. Subsequently, we employed min–max normalization, a strategy selected for its robustness with non‐Gaussian physiological data and its ability to scale disparate metrics onto a common comparable axis [32]. Application of the framework revealed that the pancreatic microcirculation in T2DM mice was impaired, but could be improved with liraglutide administration, emphasizing the adverse effects of T2DM on microcirculatory function but also suggesting the potential of liraglutide in enhancing microcirculatory function.

Our study moves beyond the conventional metric of total blood perfusion, demonstrating that stratifying flow by velocity is important for dissecting the physiology of microhemodynamic regulation [33]. The multicomponent approach reveals that T2DM selectively impairs the high‐speed (> 10 mm/s), transport‐oriented vasculature of the pancreas, a finding that contrasts sharply with reports of compromised low‐speed (< 1 mm/s), nutritive perfusion in the skin of diabetic patients [34], suggesting that diabetic microangiopathy is not a monolithic pathology but a highly tissue‐specific process. The distinct patterns of microvascular impairment reflect the architectural and metabolic demands of each organ, as well as their differential compensatory responses to the systemic diabetic milieu [35, 36].

A key feature of a healthy microvasculature is the dynamic coupling between resistance (IMR) and vasomotion, where increased rhythmic activity is physiologically linked to reduced flow opposition [37, 38, 39, 40]. A central finding of our study is that T2DM abrogates the relationship, decoupling vasomotion frequency from IMR_pancreas_ and locking the microvasculature in a pathological state of high static resistance [41, 42, 43]. We show that liraglutide reverses the functional impairment. Its pleiotropic actions on the endothelium do not simply lower IMR_pancreas_ in isolation; they re‐establish the physiological coherence between vascular resistance and dynamic regulation. The restoration of a core homeostatic axis, beyond mere improvement in bulk perfusion, provides evidence for a vasculoprotective mechanism and highlights a dimension for assessing therapeutic efficacy.

The functional integrity of the microcirculation depends on the precise coupling of hemodynamic oxygen supply to local tissue demand [44, 45, 46]. Our findings in T2DM mice reveal a decoupling of the relationship, where pancreatic blood flow becomes inefficient at maintaining tissue oxygenation. The state, evidenced by diminished C_RBC_, Hb, and SO_2_, creates a significant supply–demand mismatch that fosters a hypoxic microenvironment [47, 48]. We show that liraglutide treatment recalibrates this pathological state by restoring the coherence between blood flow and oxygen availability, suggesting its protective mechanism extends beyond simple vasodilation to promote a more efficient, nutritive perfusion that directly counteracts the hypoxia central to diabetic organ damage.

Another key finding of our study is the differential and time‐dependent impact of liraglutide compared to insulin on the pancreatic microvasculature. While both therapies improved microcirculatory parameters, their efficacy trajectories over the two‐week period were distinct. The benefits of insulin, likely indirect and secondary to the alleviation of glucotoxicity, appeared to manifest substantially by the first week, with less additional improvement in the second. In contrast, liraglutide demonstrated more progressive restoration. While significant functional benefit was evident at 1 week, attributable to acute effects like glycemic control and direct vasodilation, its superiority became more evident at the two‐week mark. Specifically, the normalization of complex parameters, for example, the coherence between oxygenated hemoglobin and speed‐resolved perfusion, and the restoration of the physiological relationship between vasomotion frequency and microvascular resistance, were established after the longer treatment duration. Temporal divergence suggests fundamentally different mechanisms. Insulin's effect, tied to metabolic normalization, may reach a functional plateau once euglycemia is approached. Liraglutide's benefits, however, appear biphasic; an initial phase of acute functional improvement is followed by a second, more cumulative phase. The latter phase likely reflects the sustained impact of its pleiotropic actions, which may lead to normalization of the pathological microenvironment and initiate beneficial vascular remodeling.

The restorative effect of liraglutide on pancreatic microhemodynamics, as observed in our study, can be attributed to its multifarious vasculoprotective actions that extend beyond glycemic control [49, 50], creating a powerful synergy of functional and structural benefits. A primary mechanism is the direct induction of endothelium‐dependent vasodilation; activation of endothelial GLP‐1 receptors by liraglutide stimulates eNOS‐mediated nitric oxide production, leading to smooth muscle relaxation, enhanced perfusion, and a direct reduction in microvascular resistance. The functional improvement is supported by liraglutide's ability to counteract the hostile pro‐inflammatory and high‐oxidative stress environment characteristic of T2DM. By suppressing key inflammatory pathways such as NF‐κB and mitigating the overproduction of reactive oxygen species [51], liraglutide reduces the expression of vascular adhesion molecules, attenuates leukocyte infiltration, and preserves endothelial integrity across multiple vascular beds [52], an effect shown to be independent of its glucose‐lowering action. Additionally, liraglutide intervenes in long‐term pathological vascular remodeling by inhibiting the proliferation and migration of vascular smooth muscle cells, alleviating calcification, and suppressing neointima formation after injury [53]. This is complemented by evidence of its capacity to promote beneficial angiogenesis in ischemic settings, potentially through the support of endothelial progenitor cells. The combination of enhanced vasodilation, systemic anti‐inflammatory action, and direct intervention in adverse cellular processes provides a mechanistic basis for the microcirculatory restoration observed in our study, positioning liraglutide as a potent vasculotropic therapy.

Several limitations of our study warrant discussion. Foremost among these is the spatial resolution of our methodology. The common microcirculatory framework provides an integrated assessment of a tissue volume and thus cannot distinguish the specific contributions of the hyper‐vascularized islet microcirculation from those of the surrounding exocrine tissue. However, given that islets receive a disproportionately high share of pancreatic blood flow, it is reasonable to infer that our signal is influenced by their functional state. As T2DM is also known to induce pathology such as fibrosis and inflammation in the exocrine pancreas, our framework captures the physiologically relevant net outcome of these combined disease processes. Future studies could overcome spatial limitations by integrating our functional framework with high‐resolution imaging modalities, such as intravital two‐photon microscopy, to enable synchronous assessment of specific microdomains, thereby resolving a key question raised by the current study. Additionally, while the invasive nature of our current approach in a murine model limits direct clinical translation, the primary translational value of our work lies in the common microcirculatory framework itself. By investigating the correlation between these peripheral microcirculatory signatures and systemic disease progression, it may be possible to identify surrogate biomarkers that reflect the health of less accessible visceral organs.

In conclusion, we establish a common microcirculatory framework for the quantitative analysis of concurrent hemodynamic and oxygenation data from pancreatic microcirculation. Applying the integrated approach to the diabetic pancreas, we show that liraglutide restores microcirculatory function by improving both blood perfusion and tissue oxygenation. Our study serves as a methodological foundation for future microvascular research and a proof‐of‐concept for using a quantitative approach to evaluate vasculotropic therapies.

## Author Contributions

M.L. and Y.L. designed the study. Y.L., B.W., X.L., X.Z., and X.Z. performed all experiments. Y.L., Y.W., M.X., W.L., and H.L. analyzed the data. M.L. and Y.L. drafted and revised the manuscript. R.X. helped to revise the manuscript. M.L. conceived and supervised the project. All authors discussed the results and commented on the manuscript.

## Funding

This work was supported by the Natural Science Foundation of Beijing Municipality (7212068) and the CAMS Innovation Fund for Medical Sciences (CIFMS: 2022‐I2M‐1‐026).

## Conflicts of Interest

The authors declare no conflicts of interest.

## Supporting information


**Data S1:** Supporting Information.

## Data Availability

Data supporting the findings of this study are available from the corresponding author upon reasonable request.
