# Supplementary material for: Algorithm‐Based Common Microcirculatory Framework for Monitoring and Visualizing the Integrated Pancreatic Microcirculation in Type 2 Diabetes Mellitus Mice
Source: J Diabetes. 2026 Feb 4;18(2):e70188. doi: 10.1111/1753-0407.70188 (PMC12869126; doi:10.1111/1753-0407.70188)
Supplement: Supplementary file 1 — Data S1: Supporting Information. [file JDB-18-e70188-s001.zip › jdb70188-sup-0001-Supinfo.docx]

**Supplementary Information**

Table S1

Table S2

Figure S1.tif

Figure S2.tif

Figure S3.tif

Figure S4.tif

Figure S5.tif

Figure S6.tif

Figure S7.tif

Figure S8.tif

Figure S9.tif

Figure S10.tif

Video S1.mp4

Video S2.mp4

Video S3.mp4

Video S4.mp4

Video S5.mp4

Video S6.mp4

Video S7.mp4

Video S8.mp4

Video S9.mp4

Video S10.mp4

Video S11.mp4

Video S12.mp4

**Additional files are available at**

https://figshare.com/s/2310e1c835eee79471bf

**TABLE S1** Information on microcirculatory oxygen and microhemodynamic parameters

| **Microcirculatory parameters** | **Unit** | **Meaning** |
| --- | --- | --- |
| C_RBC_^†^ | % | Fraction of the sampling volume that consists of RBCs |
| SO_2_ | % | C_RBC_ or Hb in the sampling volume that is saturated |
| Total Hb | μM | Amount of Hb in the sampling volume |
| Oxygenized Hb | μM | Total Hb × SO_2_ |
| Reduced Hb | μM | 1 － (Total Hb × SO_2_) |
| Total BP | % RBC×mm/s | C_RBC_ × average speed |
| Speed-resolved BP (<1 mm/s) | % RBC×mm/s | Blood perfusion with whose speed below 1 mm/s |
| Speed-resolved BP (1 – 10 mm/s) | % RBC×mm/s | Blood perfusion with whose speed at 1 – 10 mm/s |
| Speed-resolved BP (>10 mm/s) | % RBC×mm/s | Blood perfusion with whose speed above 10 mm/s |
| Conventional BP | PU | Relative blood perfusion |

Note: ^†^C_RBC_, red blood cell tissue fraction; RBC, red blood cell; SO_2_, oxygen saturation; Hb, hemoglobin concentration; BP, blood perfusion.

**TABLE S2** Two-way ANOVA comparison among pancreatic microcirculatory parameters

| **Microcirculatory parameters** | **Administration** | | | **Treatment time** | | | **Interaction** | | |
| --- | --- | --- | --- | --- | --- | --- | --- | --- | --- |
|  | *F* | *df* | *P* | *F* | *df* | *P* | *F* | *df* | *P* |
| C_RBC_^†^ | 0.6718 | 1 | 0.4165 | 3.34 | 2 | 0.0438 | 0.4994 | 2 | 0.61 |
| SO_2_ | 3.345 | 1 | 0.0736 | 5.132 | 2 | 0.0096 | 0.9458 | 2 | 0.3955 |
| Total Hb | 0.6718 | 1 | 0.4165 | 3.34 | 2 | 0.0438 | 0.4994 | 2 | 0.61 |
| Oxygenized Hb | 1.422 | 1 | 0.2389 | 5.662 | 2 | 0.0062 | 1.032 | 2 | 0.3641 |
| Reduced Hb | 0.07451 | 1 | 0.7861 | 0.9373 | 2 | 0.3987 | 0.0634 | 2 | 0.9387 |
| Total BP | 0.6718 | 1 | 0.4165 | 3.34 | 2 | 0.0438 | 0.4994 | 2 | 0.61 |
| Conventional BP | 3.616 | 1 | 0.0632 | 1.736 | 2 | 0.1871 | 0.9261 | 2 | 0.4031 |
| Speed-resolved BP (<1 mm/s) | 1.331 | 1 | 0.2543 | 1.436 | 2 | 0.2479 | 0.5389 | 2 | 0.5869 |
| Speed-resolved BP (1 – 10 mm/s) | 2.158 | 1 | 0.1484 | 1.748 | 2 | 0.1851 | 0.7159 | 2 | 0.4939 |
| Speed-resolved BP (>10 mm/s) | 2.478 | 1 | 0.122 | 0.8811 | 2 | 0.4209 | 1.146 | 2 | 0.3264 |

Note: ^†^C_RBC_, red blood cell tissue fraction; SO_2_, oxygen saturation; Hb, hemoglobin concentration; BP, blood perfusion.

**Supplementary Figure Legends**

**
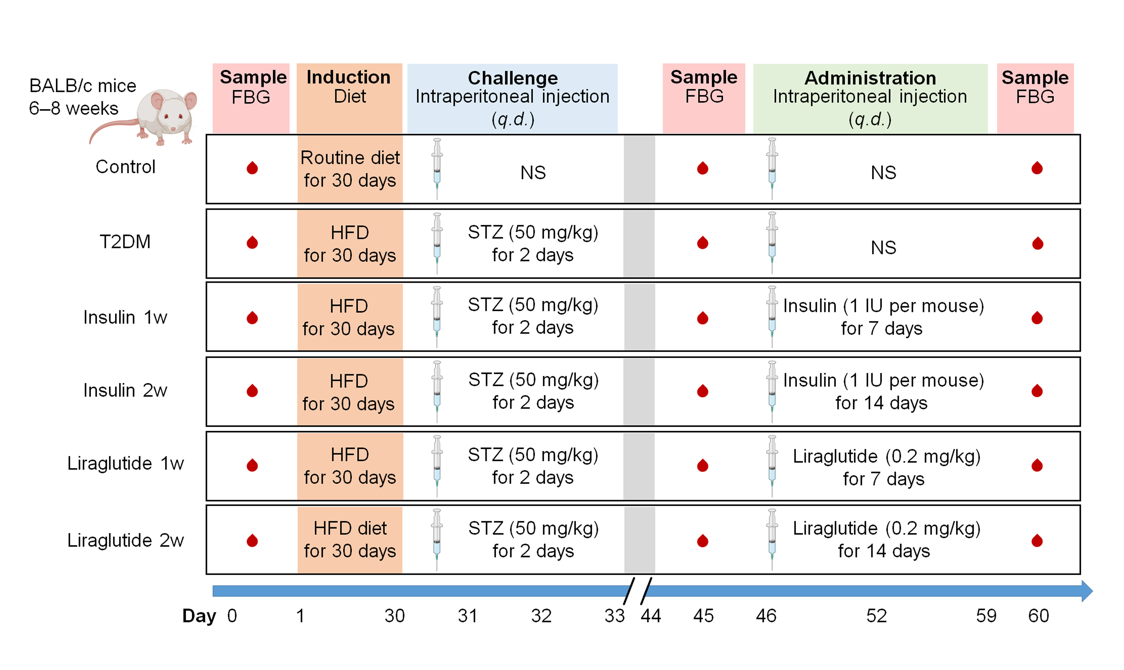
**

**FIGURE S1** Induction of T2DM, administration of insulin and liraglutide. BALB/c mice were randomly divided into control, T2DM, 1-week/ 2-week insulin-treated, and 1-week/2-week liraglutide-treated groups (*n* = 9 each group). To induce the T2DM model, mice were fed with HFD for 30 days, and then STZ was injected intraperitoneally for two consecutive days. Twelve days after the STZ injection, blood samples from the tail vein were collected and the hyperglycemia of T2DM was confirmed in mice with FBG exceeding 200 mg/dl. Mice in the control group were fed with a routine diet and challenged with normal saline at corresponding time points. Red drops represented blood sampling. FBG, fasting blood glucose; *q.d.*, once every day; HFD, high fat diet; NS, normal saline; STZ, streptozotocin; IU, international unit; T2DM, type 2 diabetes mellitus; insulin 1w, 1-week insulin-administrated T2DM group; insulin 2w, 2-week insulin-administrated T2DM group; liraglutide 1w, 1-week liraglutide-administrated T2DM group; liraglutide 2w, 2-week liraglutide-administrated T2DM group.


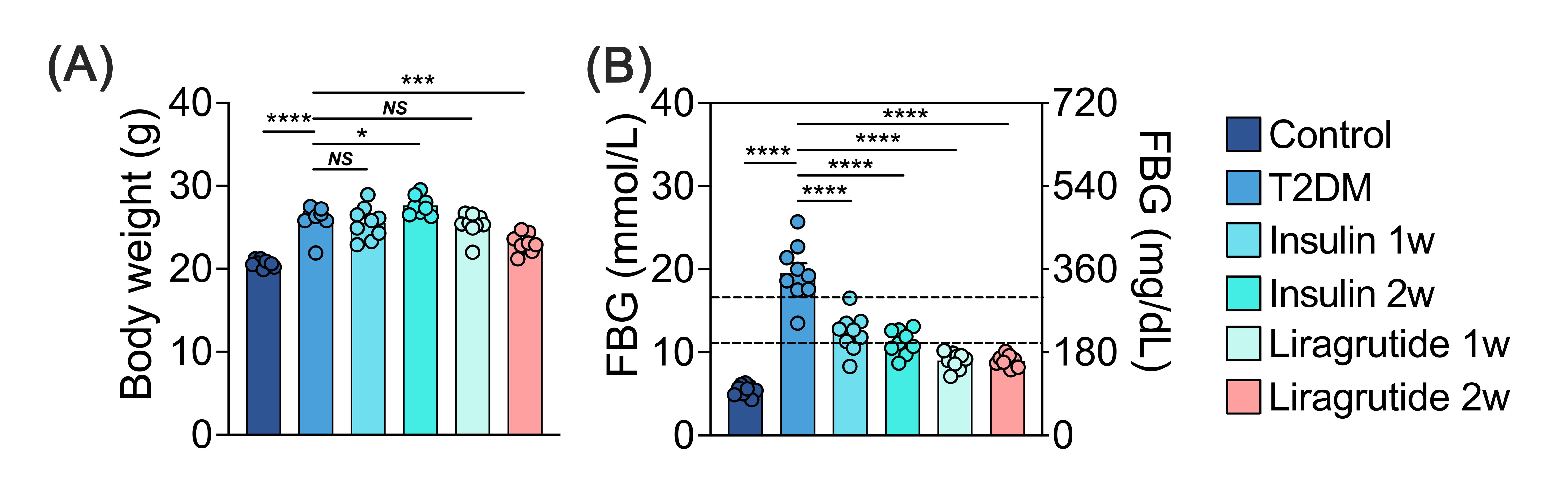


**FIGURE S2** Body weight and FBG of control, T2DM, and insulin-, liraglutide-treated groups. Body weight (A) and fasting blood glucose (B) were determined. The dotted lines upon and below represent the FBG level at 11.1 mmol/L (200 mg/dL) and 16.6 mmol/L (300 mg/dL), respectively. Values are reported as means ± standard error of the mean (SEM). * *P* < 0.05, ** *P* < 0.01, *** *P* < 0.001, **** *P* < 0.0001; *NS*, no significant difference; FBG, fasting blood glucose; HFD, high fat diet; STZ, streptozotocin; T2DM, type 2 diabetes mellitus; insulin 1w, 1-week insulin-administrated T2DM group; insulin 2w, 2-week insulin-administrated T2DM group; liraglutide 1w, 1-week liraglutide-administrated T2DM group; liraglutide 2w, 2-week liraglutide-administrated T2DM group. *n* = 9 per group.


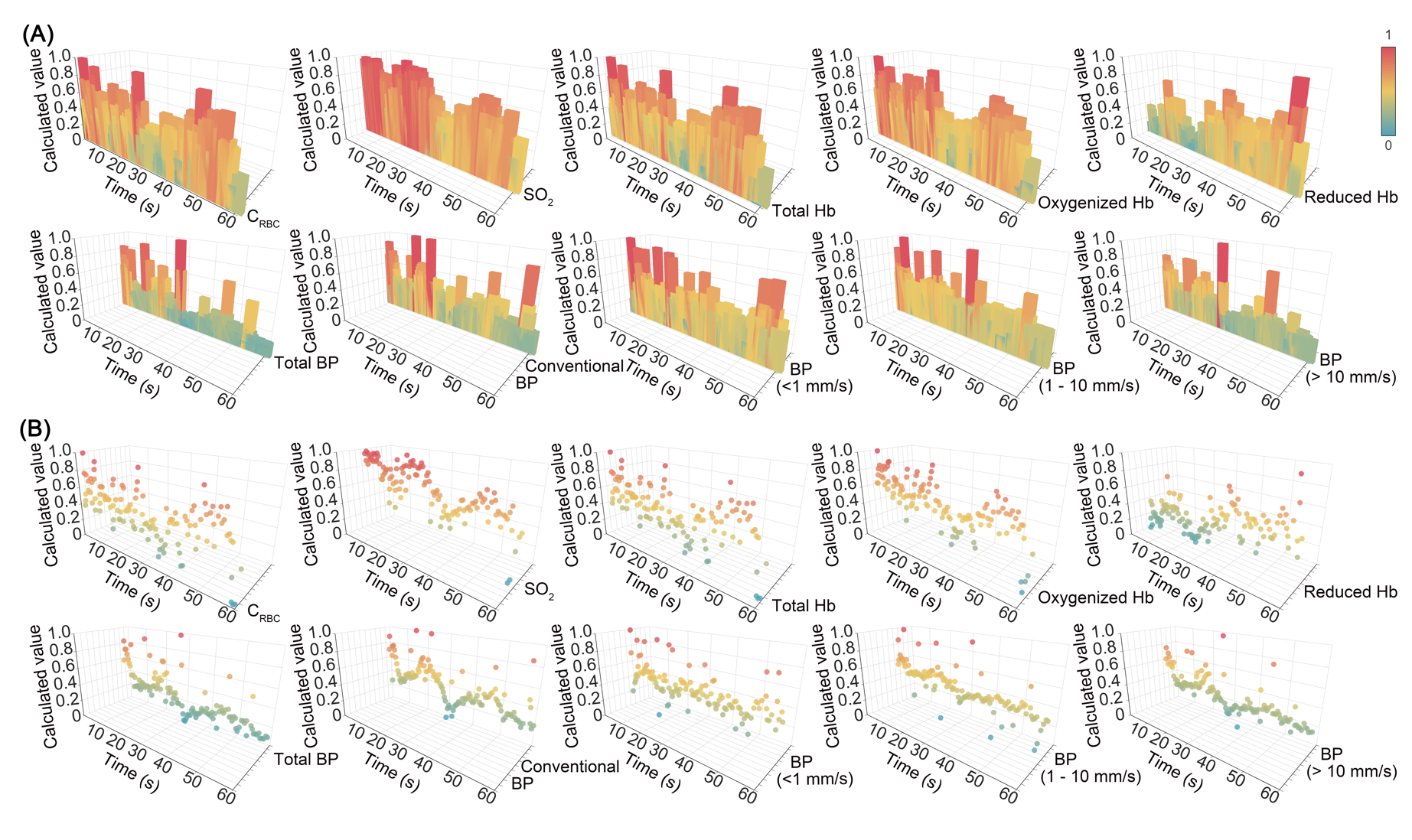


**FIGURE S3** Three-dimensional module of pancreatic microcirculatory profiles in control group.


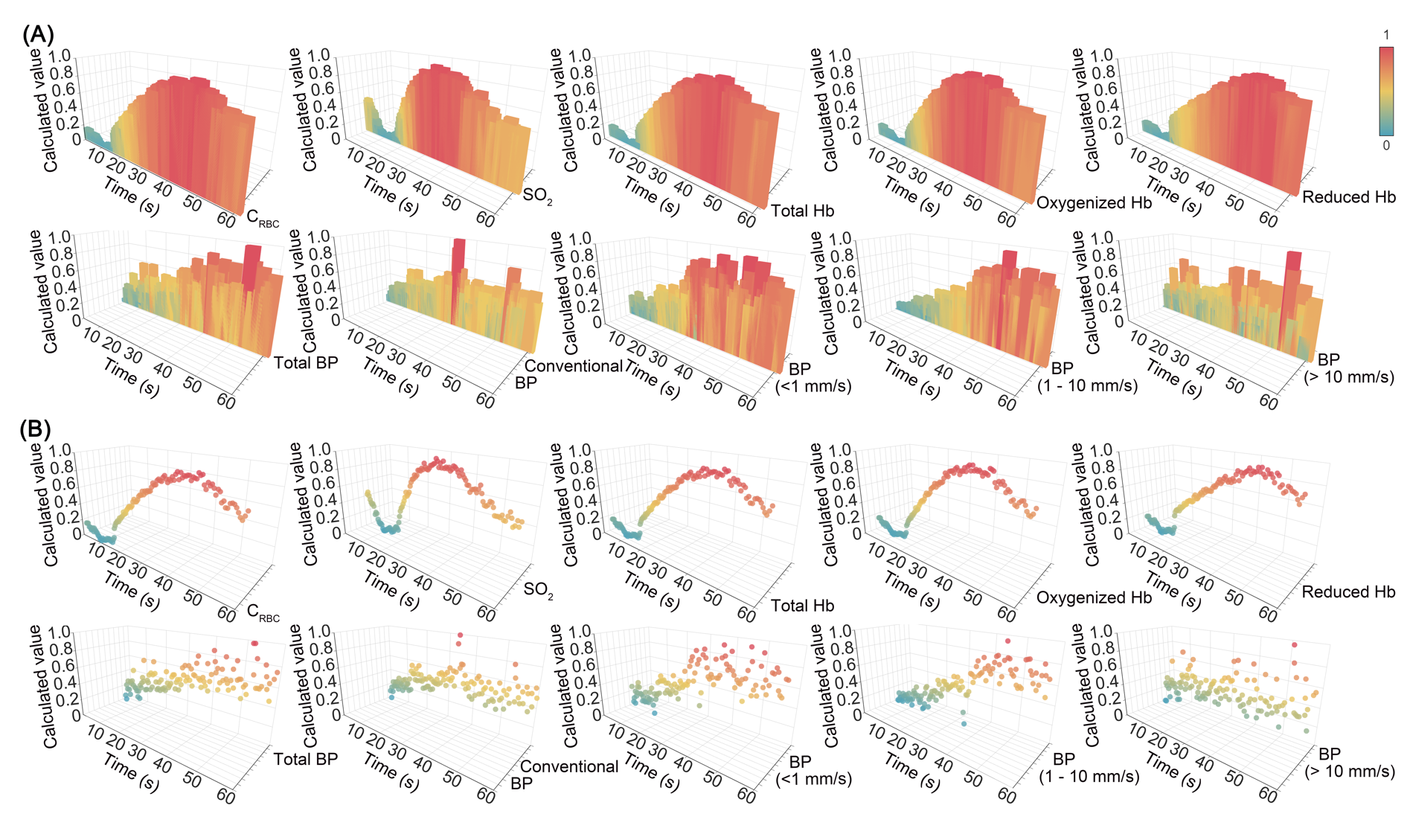


**FIGURE S4** Three-dimensional module of pancreatic microcirculatory profiles in T2DM group.

**
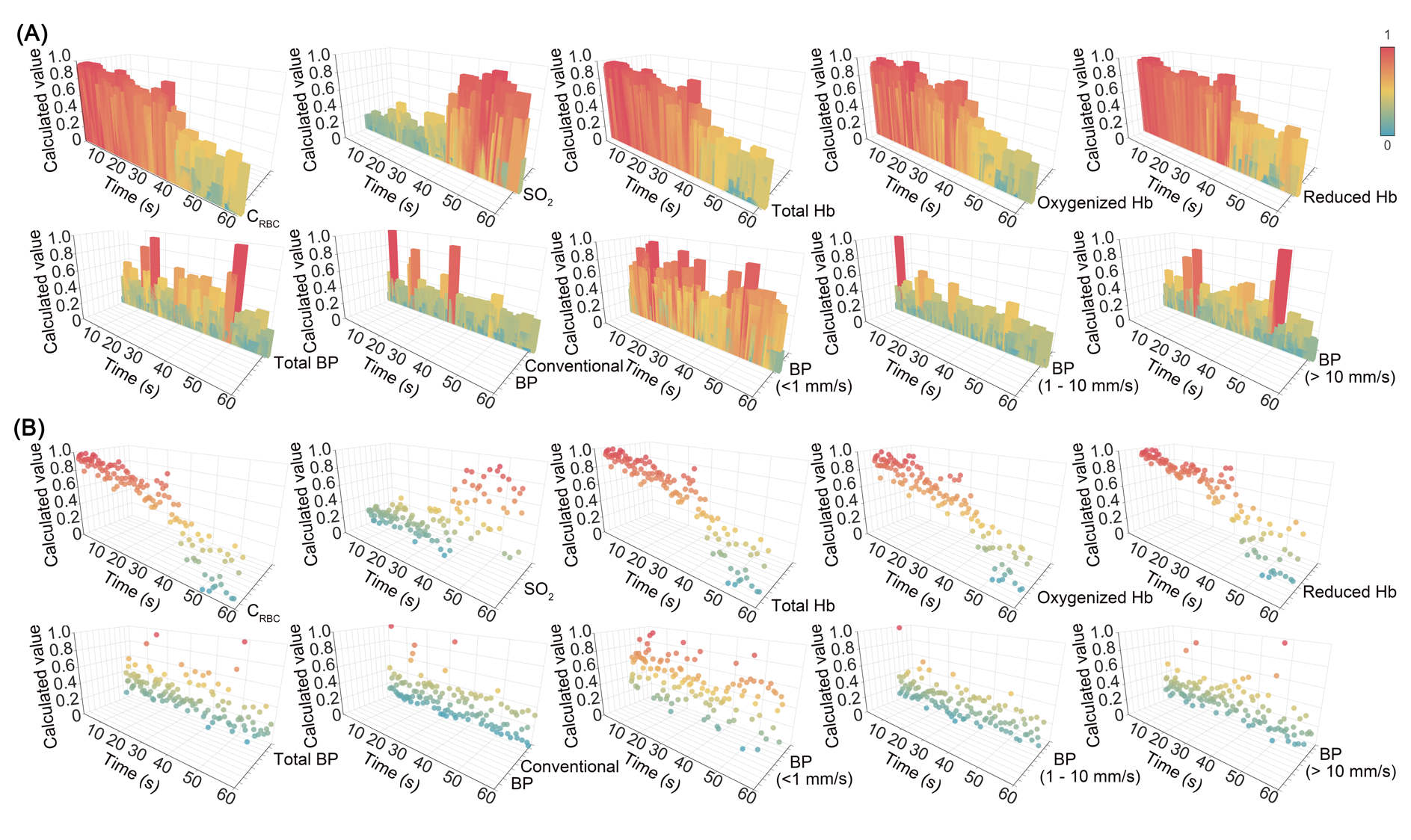
**

**FIGURE S5** Three-dimensional module of pancreatic microcirculatory profiles in one-week insulin-administrated group.


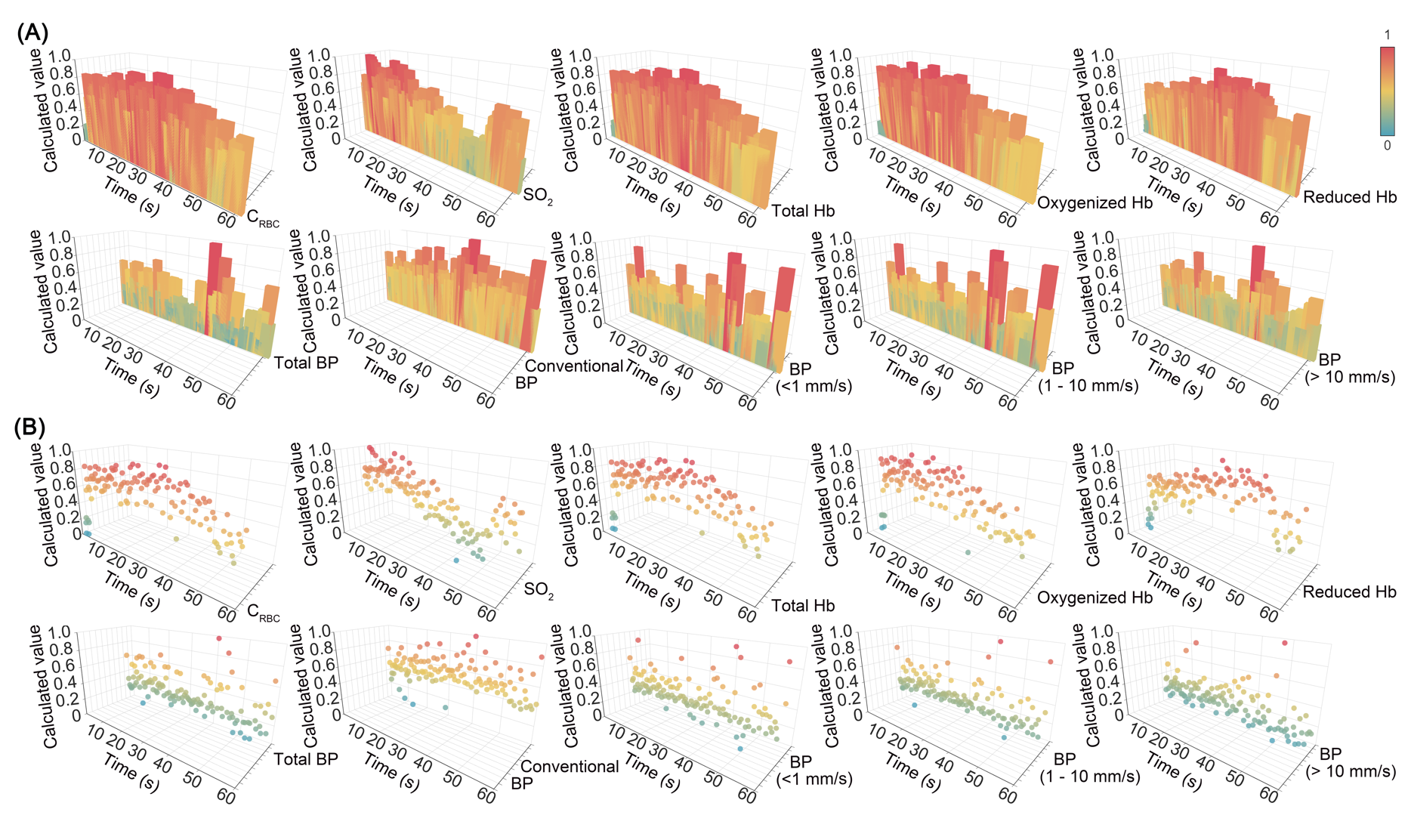


**FIGURE S6** Three-dimensional module of pancreatic microcirculatory profile in two-week insulin-administrated group.


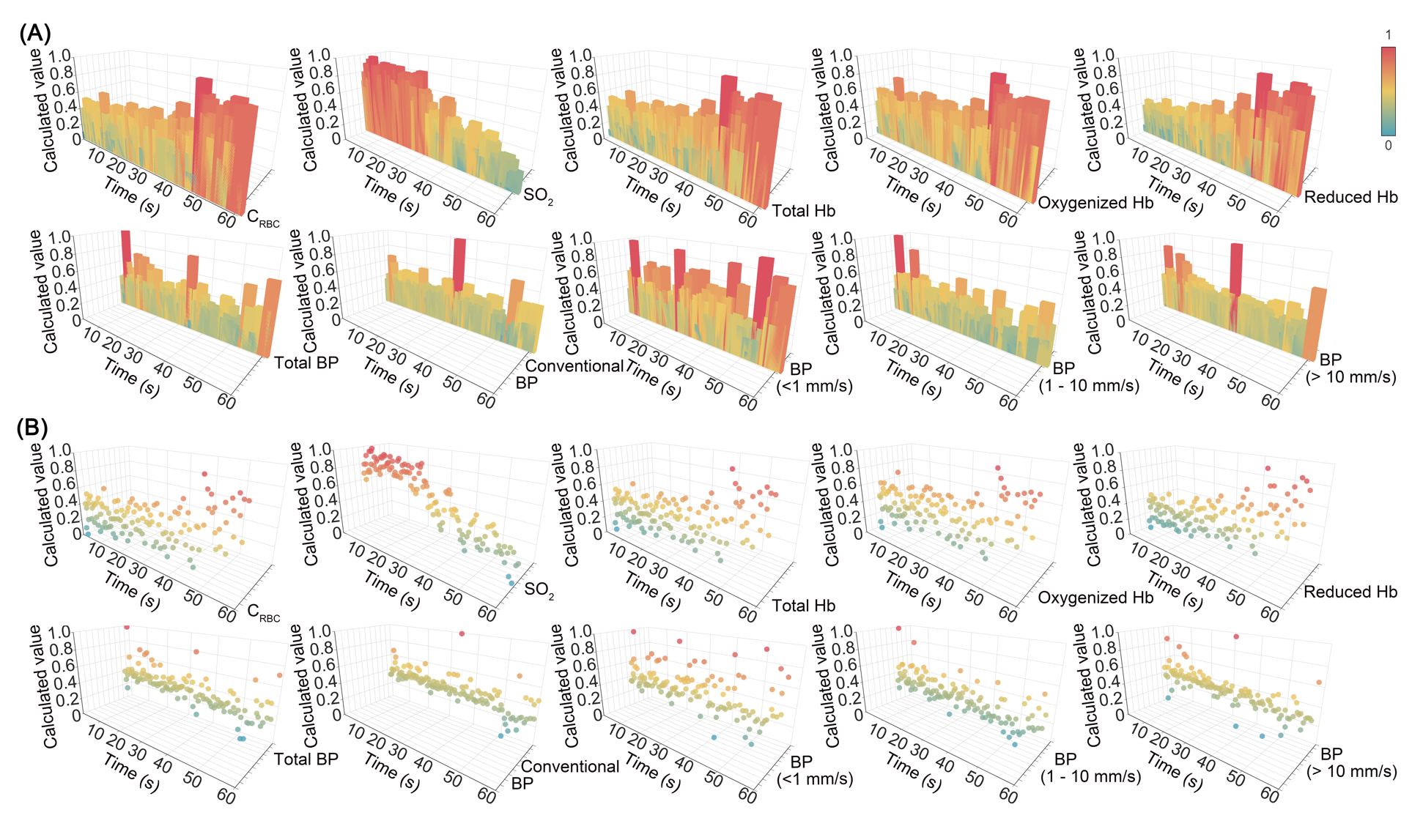


**FIGURE S7** Three-dimensional module of pancreatic microcirculatory profile in one-week liraglutide-administrated group.


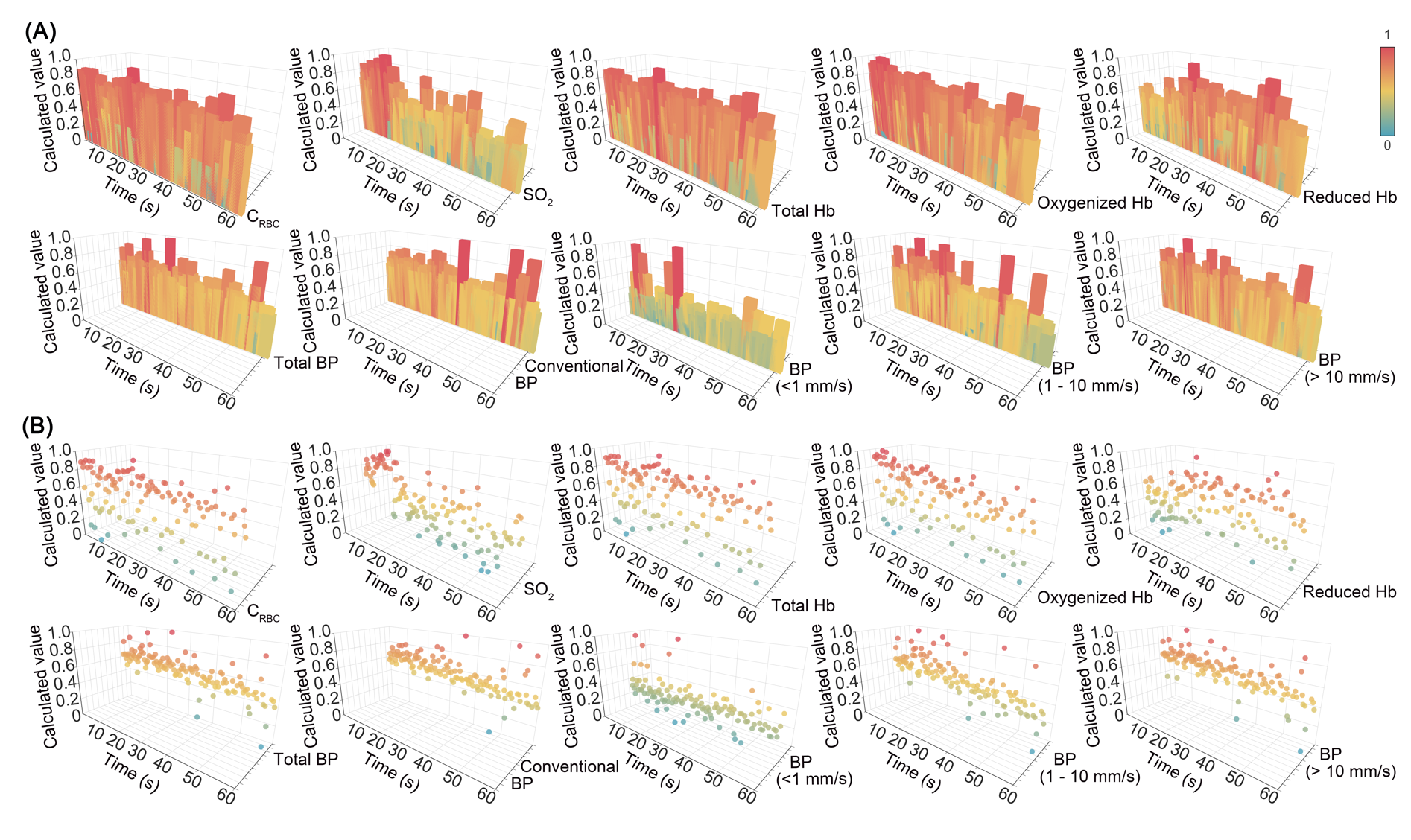


**FIGURE S8** Three-dimensional module of integrated pancreatic microcirculatory profile in two-week liraglutide-administrated group.

**FIGURE S3-8** Three-dimensional (3-D) modules of six groups were illustrated in these figures, including the control (Figure S3), T2DM (Figure S4), one-week insulin-administrated (Figure S5), two-weeks insulin-administrated (Figure S6), one-week liraglutide-administrated (Figure S7), and two-weeks liraglutide-administrated groups (Figure S8). The pancreatic microcirculatory oxygen (C_RBC_, SO_2_, total Hb, oxygenized Hb, and reduced Hb) and microhemodynamic profiles (total BP, conventional BP, and speed-resolved BP) of control mice were encapsulated within 3-D module respectively. (A) 3-D histogram module. (B) 3-D distribution module. The modules were illustrated as side view (rotated 45° toward left from the front view). Time course, pancreatic microcirculatory variables, and calculated values were defined as the X, Y, and Z axis of the 3-D module, respectively. Color bar depicts calculated values. 3-D, three-dimensional; C_RBC_, red blood cell tissue fraction; SO_2_, oxygen saturation; Hb, hemoglobin concentration; BP, blood perfusion.

**
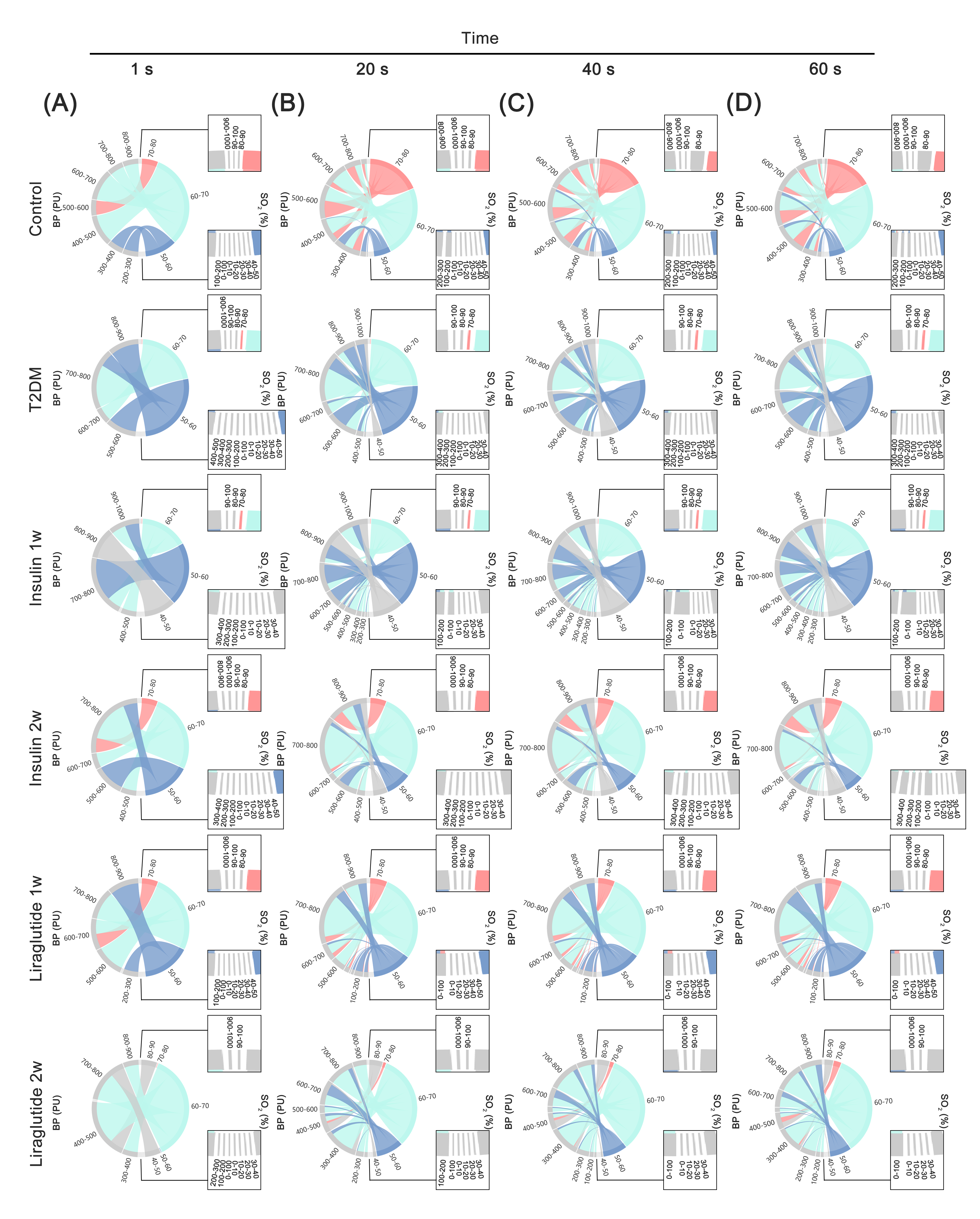
**

**FIGURE S9** Microhemodynamic-oxygenic consistency changes in time series. In order to further investigate the dynamic changes with time series between pancreatic microcirculatory blood flow and oxygen flow, microhemodynamic-oxygenic consistency in time period of 1 s (A), 20 s (B), 40 s (C), and 60 s (D) was detected among groups. The left and right arcs represented the microcirculatory blood perfusion and SO_2_, respectively. According to the microcirculatory function levels, each arc was assigned into 10 intervals. The color of blue, green, and red represent SO_2_ at 50 – 60 %, 60 – 70 %, and 70 – 80 % levels, respectively. The arc width represented the amount of microcirculatory blood perfusion and SO_2_ data, and the chord width reflected the correlation attribute between blood perfusion and SO_2_. S, second; SO_2_, oxygen saturation; BP, blood perfusion; T2DM, type 2 diabetes mellitus; insulin 1w, 1-week insulin-administrated T2DM group; insulin 2w, 2-week insulin-administrated T2DM group; liraglutide 1w, 1-week liraglutide-administrated T2DM group; liraglutide 2w, 2-week liraglutide-administrated T2DM group.


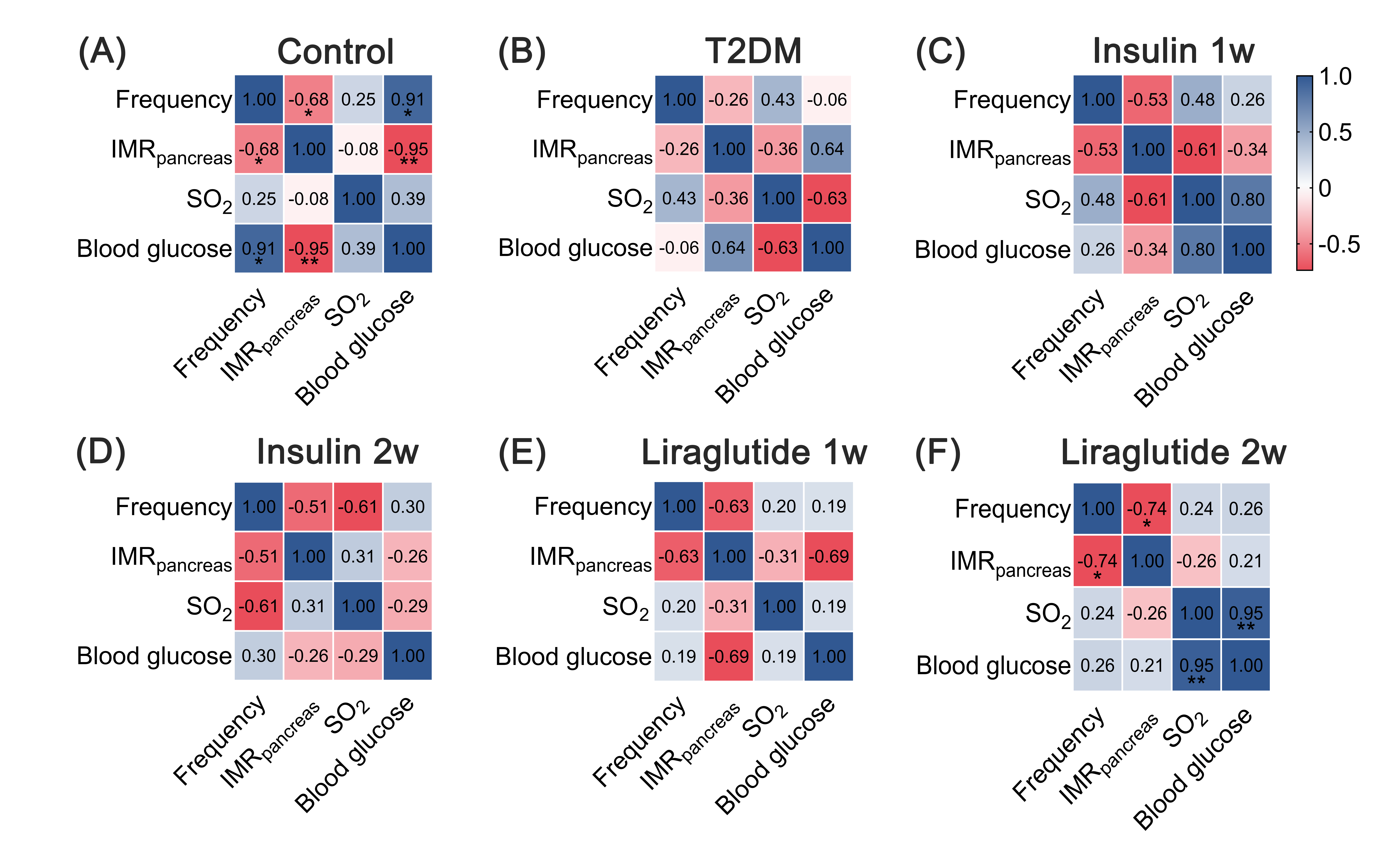


**FIGURE S10** Correlation analysis among microcirculatory profiles. (A)-(F): Heat map of microcirculatory profiles, including microcirculatory frequency, IMR_pancreas_, SO_2_, and blood glucose. The color of purple represented positive correlation, while blue represents negative correlation. (A) Control group. (B) T2DM group. (C) Insulin 1-week administrated group. (D) Insulin 2-weeks administrated group. (E) Liraglutide 1-week administrated group. (F) Liraglutide 2-weeks administrated group. **P* < 0.05, ***P* < 0.01. T2DM, type 2 diabetes mellitus; insulin 1w, 1-week insulin-administrated T2DM group; insulin 2w, 2-week insulin-administrated T2DM group; liraglutide 1w, 1-week liraglutide-administrated T2DM group; liraglutide 2w, 2-week liraglutide-administrated T2DM group; IMR_pancreas_, index of pancreatic microcirculatory resistance.

**VIDEO S1-12.** Three-dimensional (3-D) histogram and distribution modules of integrated pancreatic microcirculation profiles in six groups, including the control (Supplementary Video 1-2), T2DM (Supplementary Video 3-4), one-week insulin-administrated (Supplementary Video 5-6), two-weeks insulin-administrated (Supplementary Video 7-8), one-week liraglutide-administrated (Supplementary Video 9-10), and two-weeks liraglutide-administrated groups (Supplementary Video 11-12). Apache ECharts (version 4.2.0-rc.2) tool licensed under the Apache License (version 2.0) was used to establish the 3-D module. ScreenToGif (version 2.19.3) was employed to record the supplementary video. The video was formatted as an MP4 file. The pancreatic microcirculatory oxygen (C_RBC_, SO_2_, total Hb, oxygenized Hb, and reduced Hb) and microhemodynamic profiles (total BP, conventional BP, and speed-resolved BP) of control mice were captured and imported into constructed 3-D module. Time course, pancreatic microcirculatory variables, and calculated values were defined as the X, Y, and Z axis of the dynamic 3-D module, respectively. The established 3-D module of pancreatic microcirculation profiles allows to zoom in, zoom out, and rotate as designed. The color bar depicts calculated values. 3-D, three-dimensional; C_RBC_, red blood cell tissue fraction; SO_2_, oxygen saturation; Hb, hemoglobin concentration; BP, blood perfusion.
